# Age-related positivity effect in emotional memory consolidation from middle age to late adulthood

**DOI:** 10.3389/fnbeh.2024.1342589

**Published:** 2024-01-24

**Authors:** Xinran Niu, Mia F. Utayde, Kristin E. G. Sanders, Dan Denis, Elizabeth A. Kensinger, Jessica D. Payne

**Affiliations:** ^1^Sleep, Stress, and Memory Lab, Department of Psychology, University of Notre Dame, Notre Dame, IN, United States; ^2^Department of Psychology, University of York, York, United Kingdom; ^3^Cognitive and Affective Neuroscience Laboratory, Department of Psychology and Neuroscience, Boston College, Chestnut Hill, MA, United States

**Keywords:** emotional memory, aging, positivity effect, middle age, older adulthood, memory consolidation, memory trade-off, valence

## Abstract

**Background:**

While younger adults are more likely to attend to, process, and remember negative relative to positive information, healthy older adults show the opposite pattern. The current study evaluates when, exactly, this positivity shift begins, and how it influences memory performance for positive, negative, and neutral information.

**Methods:**

A total of 274 healthy early middle-aged (35–47), late middle-aged (48–59), and older adults (>59) viewed scenes consisting of a negative, positive, or a neutral object placed on a plausible neutral background, and rated each scene for its valence and arousal. After 12 h spanning a night of sleep (*n* = 137) or a day of wakefulness (*n* = 137), participants completed an unexpected memory test during which they were shown objects and backgrounds separately and indicated whether the scene component was the “same,” “similar,” or “new” to what they viewed during the study session.

**Results and conclusions:**

We found that both late middle-aged and older adults rated positive and neutral scenes more positively compared to early middle-aged adults. However, only older adults showed better memory for positive objects relative to negative objects, and a greater positive memory trade-off magnitude (i.e., remembering positive objects at the cost of their associated neutral backgrounds) than negative memory trade-off magnitude (i.e., remembering negative objects at the cost of their associated neutral backgrounds). Our findings suggest that while the positivity bias may not emerge in memory until older adulthood, a shift toward positivity in terms of processing may begin in middle age.

## 1 Introduction

As we approach older age, we tend to view the world more positively. Younger adults often prioritize negative information over positive information (i.e., a negativity bias; Carstensen and DeLiema, 2018; Barber and Kim, 2021), while older adults are less likely to show this bias and can even show the opposite tendency to preferentially process, attend to, and remember positive over negative information (i.e., a positivity bias). Moreover, even in the face of physical and cognitive declines, healthy aging is associated with more positive life experiences and better psychological well-being (Höglund et al., 2020; Korkmaz and Güloğlu, 2021).

Previous studies find that older adults tend to intrinsically orient their attention toward positive stimuli and away from negative stimuli (Mather and Carstensen, 2003; Rösler et al., 2005; Isaacowitz et al., 2006, 2009; Knight et al., 2007). For example, older adults display gaze preferences for happy faces but tend to look away from fearful faces (Noh and Isaacowitz, 2015; Erbey et al., 2020). Research evaluating an event-related potential (ERP) sensitive to attentional engagement to emotional information and emotion regulation, known as the late positive potential (LPP; Hajcak and Nieuwenhuis, 2006; Ferrari et al., 2008; De Cesarei and Codispoti, 2011), found that the amplitude of the LPP decreases in old age when evoked by negative stimuli, yet the LPP amplitude for positive stimuli remains invariable across the lifespan (Wood and Kisley, 2006; Kisley et al., 2007; Langeslag and van Strien, 2009). While young adults show greater LPP amplitude for negative information than positive information, older adults show comparable LPP amplitude in response to both negative and positive stimuli (Wood and Kisley, 2006; Kisley et al., 2007; Mathieu et al., 2014). These behavioral and ERP studies emphasize that the negativity bias commonly seen in younger adults diminishes as individuals age, through a modulation of attention and affective responding to negative stimuli.

The age-related positivity effect also shows up in memory. A meta-analysis demonstrated that older adults are more likely to remember positive than negative stimuli compared to younger adults (Reed et al., 2014). This positivity effect was consistently observed using incidental encoding procedures, in which participants are not explicitly instructed to remember stimuli and are unaware their memory will be tested later (Charles et al., 2003; Leigland et al., 2004; Kensinger, 2008; Piguet et al., 2008; Spaniol et al., 2008). This is likely because the positivity effect is the most robust when older adults process emotional information naturally and spontaneously during encoding.

There are several theories about why the positivity effect occurs as individuals age. The Socioemotional Selectivity Theory, for instance, states that motivations and priorities shift across the life span, such that older adults become more aware that their future time horizons are limited compared to younger adults (Carstensen et al., 1999; Carstensen and Mikels, 2005; Carstensen, 2006). Thus, while younger adults often grapple with uncertainty about their educational and occupational paths, older adults tend to act in accordance with present-focused goals that promote emotional satisfaction (Waszczuk et al., 2016). These shifts in internal goals result in the selective processing of positive over negative information (Carstensen et al., 1999; Carstensen and Mikels, 2005; Carstensen, 2006). In contrast, other theories propose that the positivity effect results from age-related cognitive or neural deficits. The Dynamic Integration Theory states that due to older adults’ natural cognitive decline, they prioritize the processing of positive more than negative information because negative information tends to be more complex (Labouvie-Vief et al., 2010). Relatedly, the aging brain model proposes that the positivity effect arises as a result of amygdala degradation in old age. Given the importance of the amygdala in evaluating and responding to negative emotional information, amygdala degradation may lead to decreased sensitivity to negative information, therefore resulting in a comparative increase in the processing of positive information (Anderson, 2007; Sergerie et al., 2008).

Contrary to the Dynamic Integration Theory and the aging brain model theory, however, some imaging studies observe structural and functional preservation of the amygdala in normal aging, which explains why the amygdala’s responsiveness to *positive* stimuli does not vary across age (Allen et al., 2005; Leclerc and Kensinger, 2008; Brabec et al., 2010). Though some fMRI studies do find less amygdala activity in older adults while viewing negative information in comparison to their younger counterparts, these studies concurrently observe higher medial prefrontal cortical activity, which may indicate increased recruitment of cognitive control to regulate negative emotions (Mather et al., 2004; Williams et al., 2006; St Jacques et al., 2009; Leclerc and Kensinger, 2011; Samanez-Larkin and Carstensen, 2011). Therefore, increased cognitive control during emotional processing in old age, suggested by increased PFC activation, may underlie the positivity effect, rather than cognitive deficits in processing complex information or impaired amygdala function. Taken together with the aforementioned ERP findings suggesting that attentional engagement to negative but not positive information decreases with age (Wood and Kisley, 2006; Kisley et al., 2007; Langeslag and van Strien, 2009), older adults may show a more deliberate and controlled form of affective regulatory activity that maintains the emotional processing of positive memories while diminishing the processing of negative memories (Nashiro et al., 2011).

Although increasing age is associated with remembering positive experiences better than negative ones, both positive and negative experiences are disproportionately represented in memory compared to neutral ones (McGaugh, 2000; Christianson, 2014; Williams et al., 2022). This is in part due to the influence of emotional arousal at the time of encoding, which triggers the release of stress-related neuromodulators, such as norepinephrine and cortisol, which “tag” emotional aspects of memories that are worth retaining for future recollection (Holland and Kensinger, 2010; Bennion et al., 2015; Payne and Kensinger, 2018). One of the paradigms that has consistently found enhancements in emotional memory is the emotional memory trade-off task, where recognition memory is assessed separately for objects and backgrounds within scenes containing either an emotional or neutral object placed on a neutral background (Payne et al., 2008, 2012, 2015; Payne and Kensinger, 2011; Chambers et al., 2014; Bennion et al., 2017; Alger et al., 2018; Cummings et al., 2018; Vargas et al., 2019; Denis et al., 2022). Studies using this task consistently demonstrate that participants exhibit better memory for emotional objects than any other type of memory measured by the task, which includes neutral objects and the (also neutral) backgrounds paired with either type of object (Payne et al., 2008, 2012, 2015; Payne and Kensinger, 2011; Chambers et al., 2014; Bennion et al., 2017; Alger et al., 2018; Cummings et al., 2018; Vargas et al., 2019; Denis et al., 2022). Previous studies also find that sleep further enhances this emotional memory trade-off effect, potentially because sleep is a critical stage where emotionally salient information is preferentially consolidated (Payne et al., 2008, 2012; Payne and Kensinger, 2011; Cunningham et al., 2014; Bennion et al., 2015, 2017; Denis et al., 2022; Sanders et al., n.d.).

Importantly, however, most prior studies using the emotional memory trade-off task focus on emotional memory for negatively valenced content, with only a few studies examining positive stimuli (Chambers et al., 2014; Denis et al., 2022). Negative stimuli are often used because negative information tends to elicit higher levels of emotional arousal than positive information, resulting in a stronger effect on memory (Kensinger, 2009; Kauschke et al., 2019; Cook et al., 2023). For example, a vicious looking snake might be perceived as more unpleasant and agitating than a cute puppy is perceived as pleasant and exciting, and thus is more likely to be remembered. However, since positive episodic memories are crucial protective factors against adverse mental health outcomes, it is important to clarify whether there is also a memory trade-off effect for positive information (i.e., whether memory for positive emotional content can also be enhanced at the expense of neutral features of the same event; Holland and Kensinger, 2010; Williams et al., 2022). Moreover, knowing when this positivity shift emerges in adulthood can provide insight into the cognitive-affective mechanisms involved in other key psychological functions, including emotional regulation, decision-making, and psychological well-being as we age (Shohamy and Daw, 2015; Isaacowitz, 2022).

While the age-related positivity effect and emotional memory trade-off effect have been well researched separately, whether and how the positivity effect might interact with the emotional memory trade-off across the age-span remains relatively unexplored. It is unclear whether the age-related positivity effect is so strong that fewer attentional resources would be allocated to neutral features of the same scene, eventually leading to impaired memory performance of the peripheral neutral information in the background (Easterbrook, 1959; Reisberg and Heuer, 2004). Moreover, while it is well established that older adults tend to remember positive over negative information as mentioned above (Reed et al., 2014), it is not yet known if the neutral peripheral background of the attention-capturing object is more poorly remembered when the central event is positive compared to negative. Finally, most prior studies on the age-related positive memory bias have tested memory following brief delays, often because they were focused on how the preferential allocation of attentional resources during encoding leads to superior memory for positive information (Barber and Kim, 2021). As a result, delayed memory tasks that capture consolidation-related processes have not been widely employed.

The current study seeks to investigate the effects of aging on the selective consolidation of emotionally positive and negative central objects at the potential cost of their associated neutral backgrounds. We examined this question using a large sample of middle-aged and older adults (*n* = 274). Because we were interested in whether consolidation processes would influence these variables, we tested recognition memory after a significant delay (*M* = 11.86 h). This experiment is part of a larger study examining the impact of sleep (vs. wakefulness) on memory. As such, the memory delay interval spanned either a night of sleep or a day of wakefulness. However, because we were less interested in the specific impact of sleep here and more interested in the impact of a consolidation delay (i.e., the general passage of time), we collapsed these groups for maximum power. We hypothesized that increasing age would be associated with positive and neutral (but not negative) information being rated more positively, and with better memory performance following the delay for neutral and positive (but not negative) objects at the cost of associated neutral backgrounds. Although we did not have a firm prediction about when in the age span these positivity biases would emerge, we suspected that they would arise sometime in late middle age or early older age.

## 2 Methods

### 2.1 Participants

A total of 554 eligible participants were recruited online via Prolific (https://www.prolific.co). Inclusion criteria for eligible participants were (1) be at least 35 years of age, (2) be currently residing in the United States, (3) be fluent in English, (4) have normal or corrected-to-normal vision, and (5) have no history of any diagnosed sleep, psychiatric, or neurological disorders. Of the eligible participants, 277 completed the study. However, three were excluded due to noncompliance with the study instructions (i.e., did not rate the valence or arousal levels of any scenes). Therefore, our final sample consisted of 274 participants (*M*_age_ = 55.57, SD_age_ = 12.15). An *a priori* power analysis indicated that a sample size of 280 participants was sufficient to detect group differences (Cohen’s *d* = 0.33) between the daytime wake and nighttime sleep conditions with 80% power and α = 0.05 (Denis et al., 2022). A *post hoc* power analysis showed that a sample size of 274 participants yielded 80% power for detecting age by valence interaction (Cohen’s *f* = 0.21) with α = 0.05.

The majority of the sample self-identified as white (86.9% White, 6.2% Black/African American, 5.8% Asian, 0.7% American Indian/Native Alaskan, 0.0% Native Hawaiian/Pacific Islander, and 0.4% other; 4.0% Hispanic/Latino/Spanish). Approximately half of the sample reported their biological sex as female (51.8% female, 48.2% male, and 0% intersex). Median annual household income was 65,000 U.S. dollars (range: 0 to 300,000). See Supplementary Table S1 for a detailed breakdown of participant demographic characteristics based on age groups and delay conditions. Procedures were approved by the Institutional Review Board at the University of Notre Dame. Participants gave written informed consent and received compensation through Prolific payments for their participation.

### 2.2 Materials

#### 2.2.1 Emotional memory trade-off task

**Figure 1:**
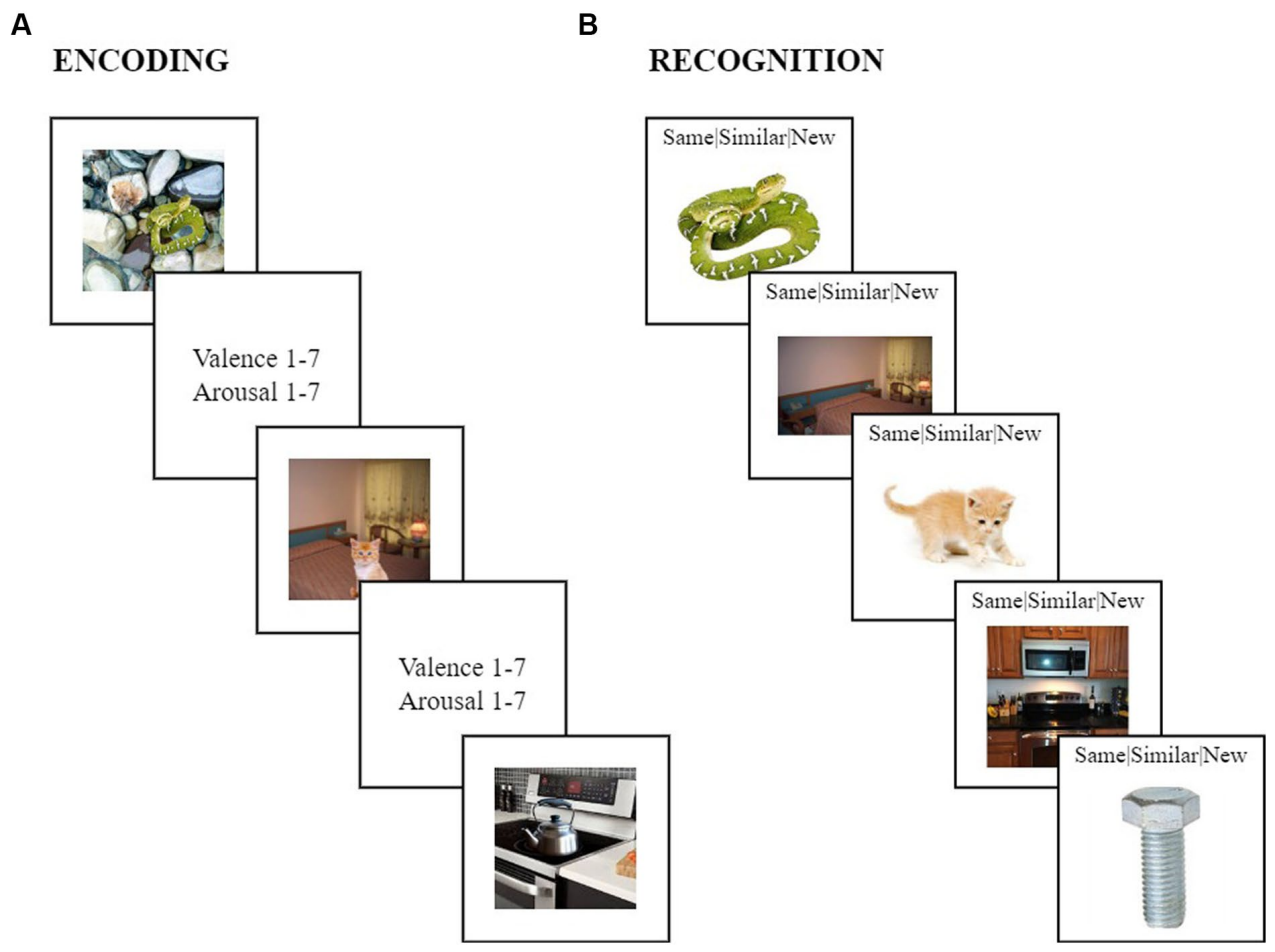
The emotional memory trade-off task. **(A)** During encoding, participants viewed a series of negative (e.g., a vicious looking snake on wet pebbles), positive (e.g., a cute kitten on a bed) and neutral scenes (e.g., a kettle on a kitchen stove), consisting of objects with negative, positive, or neutral valences always placed on a neutral background. Participants rated the valence and arousal levels of each scene on a scale from one to seven. **(B)** During recognition, participants indicated whether each scene component was “same” (e.g., the *same* snake exactly matched what has been viewed during encoding), “similar” (e.g., the *similar* kitten shared the same verbal label as the kitten during encoding but differed in specific visual details), or “new” (e.g., the bolt was *new* and was not seen during encoding).

Without being informed about the subsequent memory task, participants viewed a series of 72 scenes in a random order, each shown for 5 s. These scenes stayed the same across all participants, and depicted 24 emotionally negative, 24 positive, and 24 neutral objects, which were always placed on a plausible neutral background (Figure 1A). After each scene was presented, participants had 10 s to rate the scene for its valence (1 = highly negative, 4 = neutral: neither negative nor positive, and 7 = highly positive) and arousal levels (1 = highly calming/subduing, 4 = neither agitating/exciting nor calming/subduing, and 7 = highly agitating/exciting). Previous studies using the same stimuli found that negative scenes were rated as more negative and arousing compared to both positive and neutral scenes, and positive scenes were rated as more positive than negative and neutral scenes, and more arousing than neutral scenes (Sanders et al., n.d.).

While object and background components were presented together within intact scenes during incidental encoding, objects and backgrounds were shown separately during a surprise memory test (Figure 1B). Specifically, the recognition test consisted of 204 images presented in a random order, each displayed for 10 s. While each image was displaying, participants indicated whether it was “same,” “similar,” or “new” compared to what they had encountered during encoding. Of the 204 images (i.e., objects and backgrounds), 72 were exactly the same as participants had encountered during encoding (*same*), another 72 shared the same verbal labels as those encountered during encoding but had some variations in visual details (*similar*), and an additional 60 images were never encountered by participants during encoding (*new*). All three image sets included 12 negative objects, 12 positive objects, and 12 neutral objects. The *same* and *similar* sets were composed of 36 neutral backgrounds, whereas the *new* set contained 24 neutral backgrounds. Participants were never presented with both the *same* and *similar* versions of a particular item during the recognition test, but instead, the presentation of *same* and *similar* versions were counterbalanced across participants.

### 2.3 Procedure

Participants enrolled in a study titled “Emotional Reactivity at Different Times of Day” via Prolific (https://www.prolific.co), which redirected them to an initial screening survey. In the screening survey, participants provided information regarding their demographic characteristics and sleep. Eligible participants were then randomly assigned to either a nighttime sleep or daytime wake condition. For participants in the nighttime sleep condition, session one occurred in the evening of the same day as they completed the screening survey, between 7 and 11 p.m. in their local time, and session two was scheduled for the following morning, between 7 and 11 a.m. in their local time. In contrast, participants in the daytime wake condition performed session one in the morning (7–11 a.m.) of the next day, and session two in the evening of the next day (7–11 p.m.).

During session one, all participants completed questionnaires assessing their subjective sleep and well-being, including a three-night sleep log where participants provided information on the approximate times they went to bed and woke up, how long it took them to fall asleep, and the number of awakenings during the night for the three nights preceding the study. After completing these questionnaires, participants performed a brief 3-min version of the Psychomotor Vigilance Test (Basner et al., 2011). Then, they were presented with the series of 72 scenes, each featuring a negative, positive, or neutral object placed on an (always) neutral background. Participants were instructed to rate the valence and arousal levels of each scene. Participants had the option to take a self-paced break in the middle of the scene viewing task. After the task, they responded to additional questionnaires on their sleep and well-being. Importantly, they were not informed about the later memory task, and instead were told they would take part in another scene viewing task for session two.

After an approximately 12-h consolidation delay, participants began session two of the study. All participants completed questionnaires on sleep and well-being. Participants in the nighttime sleep condition additionally answered questions on how they slept between session one and session two. Then, all participants performed another brief version of the Psychomotor Vigilance Test. Next, they viewed a series of 108 objects and 96 backgrounds, and determined if each of the objects and backgrounds was “same,” “similar,” or “new” to what they had encountered during the encoding session. The memory test was divided into three sections to mitigate fatigue, and participants were given the option to take a self-paced break between each section. After the memory task, participants responded to additional questionnaires on sleep and well-being.

The scene rating and memory tasks were programmed in jsPsych (de Leeuw, 2015) and hosted on Cognition.run (https://www.cognition.run). All questionnaires were created and administered on Qualtrics (https://www.qualtrics.com). Refer to Supplementary material for a comprehensive list of measures for sleep, alertness, and well-being.

### 2.4 Analyses

All data processing and analyses were carried out in R (R Core Team, 2022). For all analyses, we performed two Analyses of Variance (ANOVAs), treating age as both a three-tier category (early middle age: 35–47, late middle age: 48–59, older: 60 and older) and a continuous variable (range: 35–92). The selection of age cutoffs for early middle-aged, late middle-aged, and older adults was made to maintain consistency with other studies conducted by the same research group for cross-experiment comparisons (Denis et al., 2022; Sanders et al., n.d.). Effect sizes were estimated using partial eta-squared (ηp^2^) for ANOVA models and Cohen’s *d* for follow-up *t*-tests. We report both the unadjusted, original *p*-values and *p*-values controlled for the false discovery rate (FDR; Benjamini et al., 2001). The threshold for statistical significance was set to FDR-adjusted *p* < 0.05, two-tailed.

#### 2.4.1 Valence and arousal

To investigate when the impact of age-related positivity effects on valence and arousal ratings (i.e., positive interpretation bias) emerges in adulthood, we conducted a mixed-model ANOVA with a 3 (Age: Early Middle Age, Late Middle Age, Older Adulthood) × 3 (Valence: Negative, Neutral, Positive) x 2 (Delay: Sleep, Wake) design, for valence and arousing ratings during encoding sessions. We ran pairwise *t* tests to compare valence and arousal ratings across the following conditions (1) negative, positive, and neutral scenes, (2) negative, positive, and neutral scenes within each age group, and (3) early middle-aged, late middle-aged, and older adults for each scene type. Additionally, we also performed a linear mixed-effects ANOVA, treating age as a continuous variable, and assessed the Pearson’s correlations between age and valence/arousal ratings for each scene type.

#### 2.4.2 Recognition memory

For the emotional memory trade-off task, we scored both “specific memory,” which emphasizes the retention of detailed visual properties of each component, and “gist memory” which captures meaning-based general properties of individual scene component (Reyna, 2012). For gist memory, which we focused on in this manuscript, hit rates were calculated by the proportion of trials in which participants responded with “same” or “similar” when they encountered *same* items. In contrast, specific memory was assessed more stringently, with a hit constituting only “same” response for a *same* item. False alarm rates for gist and specific memory were computed using the same methods, which was the proportion of trials in which participants incorrectly responded with “same” when encountering new items. To obtain corrected recognition memory, false alarm rates were subtracted from hit rates, yielding the final gist and specific memory scores.

To examine whether and when age-related positively effects extend to memory, we performed a 3 (Age: Early Middle Age, Late Middle Age, Older Adulthood) × 3 (Valence: Negative, Neutral, Positive) × 2 (Component: Object, Background) × 2 (Delay: Sleep, Wake) mixed-model ANOVA, as well as a linear mixed-effects ANOVA treating age as a continuous variable, for both gist and specific memory. *T*-tests were conducted to examine pairwise comparisons in gist and specific memory across the following conditions, both for the total sample and within each age group: (1) negative, positive and neutral objects, (2) neutral backgrounds associated with negative, positive, and neutral objects, and (3) objects and backgrounds within each scene type. To assess age-related differences in gist and specific memory, we carried out follow-up *t* tests to compare memory performance between early middle-aged, late middle-aged, and older adults and tested Pearson’s correlations between age and memory for all six scene components. To test how valence and arousal ratings during encoding influence memory performance, we conducted six separate multiple regression analyses. Each regression model included both valence and arousal ratings as predictors on a continuous scale, predicting memory for one of the six scene components.

#### 2.4.3 Memory trade-off effect

We calculated a memory trade-off magnitude as corrected recognition memory for (positive, negative, and neutral) objects minus corrected recognition memory for their paired neutral backgrounds during encoding, for both gist and specific memory. A positive memory trade-off magnitude indicates better memory for objects at the cost of their associated backgrounds, a magnitude of zero signifies equal memory performance between objects and backgrounds, while a negative magnitude reflects better memory for backgrounds at the expense of objects.

To analyze whether and when age-related positivity effects on memory trade-offs emerge in adulthood, we conducted a mixed-model ANOVA with a 3 (Age: Early Middle Age, Late Middle Age, Older Adulthood) × 3 (Valence: Negative, Neutral, Positive) × 2 (Delay: Sleep, Wake) design, respectively for the gist and specific memory trade-off measures. We followed-up these ANOVAs with pairwise *t*-tests across the following conditions: (1) negative, positive, and neutral scenes, (2) negative, positive, and neutral scenes within each age group, and (3) early middle-aged, late middle-aged, and older adults for each scene type. We also tested age as a continuous predictor in linear mixed-effects ANOVA, and assessed age-related correlations with gist and specific memory trade-off magnitudes for negative, positive, and neutral scenes. Finally, to test how valence and arousal ratings during encoding influence memory trade-off effects, we conducted three separate multiple regression analyses, using valence and arousal ratings as predictors in each model for memory trade-off effects on negative, positive, and neutral scenes, respectively.

## 3 Results

### 3.1 Valence and arousal

#### 3.1.1 Main effect of valence

Treating age as a categorical predictor, we found a significant main effect of scene valence on both valence [*F*(2, 536) = 1906.28, *p* < 0.001, adjusted *p <* 0.001, ηp^2^ = 0.88] and arousal ratings during encoding [*F*(2, 536) = 480.86, *p* < 0.001, adjusted *p* < 0.001, ηp^2^ = 0.64]. The main effects on valence [*F*(2, 540) = 59.77, *p <* 0.001, adjusted *p <* 0.001, ηp^2^ = 0.18] and arousal [*F*(2, 540) = 9.90, *p <* 0.001, adjusted *p <* 0.001, ηp^2^ = 0.04] were also significant when treating age as a continuous predictor. Follow-up *t*-tests were all highly significant in the expected directions (Supplementary Table S4).

#### 3.1.2 Age × valence interaction

**Figure 2:**
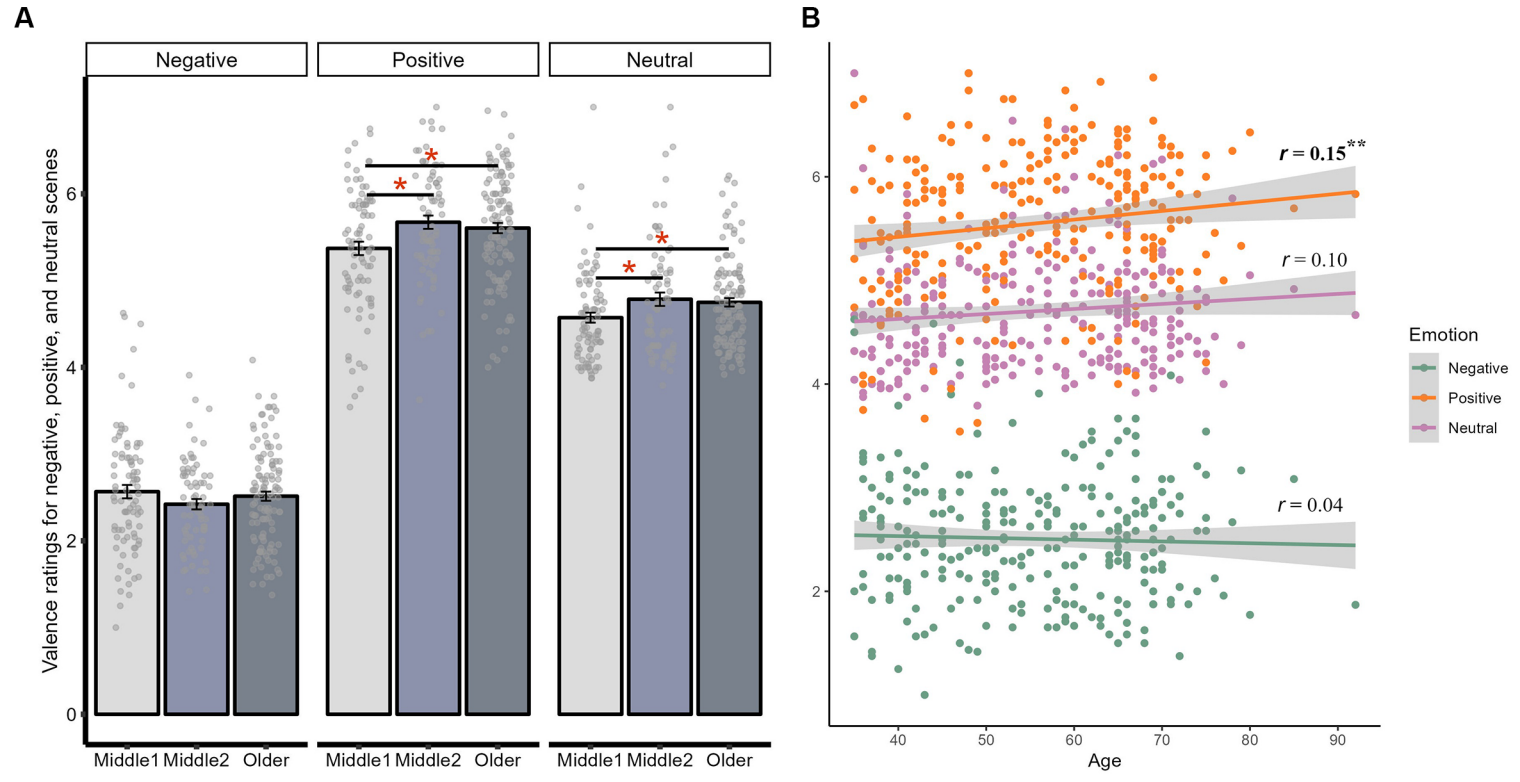
Interaction between age and valence on valence ratings. **(A)** The average valence ratings for negative, positive, and neutral scenes within early middle-aged, late middle-aged, and older adults. **(B)** The correlation between age and valence ratings for negative, positive, and neutral scenes. **p < 0.01; *p < 0.05.

We also found an interaction between age and scene valence on valence ratings, although this interaction only reached significant when age was considered as a categorical [*F*(4, 536) = 3.38, *p =* 0.010, adjusted *p =* 0.035, ηp^2^ = 0.03], and was marginal when age was considered as a continuous variable [*F*(2, 540) = 2.98, *p =* 0.052, adjusted *p =* 0.120, ηp^2^ = 0.01]. Compared to early middle-aged adults, late middle-aged [*t*(156) = 2.77, *p =* 0.006, adjusted *p =* 0.019, *d =* 0.44] and older adults [*t*(170) = 2.41, *p =* 0.017, adjusted *p =* 0.026, *d =* 0.35] both rated positive scenes as more positive. Older age was also significantly correlated with rating positive scenes more positively (*r* = 0.15, *p =* 0.013, adjusted *p =* 0.039). Similarly, late middle-aged [*t*(138) = −2.21, *p =* 0.029, adjusted *p =* 0.044, *d =* −0.35] and older adults [*t*(181) = −2.35, *p =* 0.020, adjusted *p =* 0.044, *d =* −0.34] also rated neutral scenes more positively compared to early middle-aged adults, although older age was not significantly correlated with more positive ratings for neutral scenes (*r* = 0.10, *p =* 0.088, adjusted *p =* 0.132). In summary, while there was no age-related difference in valence ratings for negative scenes, as age increased, there was an increased positive interpretation bias for both positive and neutral scenes (Figure 2).

The interaction between age and valence on arousal ratings, whether looking at age as a categorical [*F*(4, 536) = 2.79, *p =* 0.026, adjusted *p =* 0.091, ηp^2^ = 0.02] or continuous predictor [*F*(2, 540) = 3.91, *p =* 0.021, adjusted *p =* 0.072, ηp^2^ = 0.01], was only significant before adjusting for multiple comparisons. None of the pairwise age comparisons or age-related correlations were significant either (*ps* > 0.8, Supplementary Tables S4, S14). Notably, although both early [*t*(85) = 3.27, *p =* 0.002, adjusted *p =* 0.002, *d =* 0.28] and late middle-aged adults [*t*(72) = 2.78, *p =* 0.007, adjusted *p =* 0.007, *d =* 0.29] perceived positive scenes as more arousing than neutral scenes, this effect was not significant for older adults [*t*(114) = 0.81, *p =* 0.421, adjusted *p =* 0.421, *d =* 0.06]. However, the correlation between age and perceived arousal of positive information was not significant (*r* = 0.10, *p =* 0.088, adjusted *p =* 0.132).

### 3.2 Gist memory

We focused on gist memory given its resilience against age-related cognitive deficits and memory delay, especially over extended delay periods (Davis et al., 2003; Titz and Verhaeghen, 2010; Matorina and Poppenk, 2021). Results for specific memory mostly followed the same patterns as for gist memory (see Supplementary material).

#### 3.2.1 Valence × component interaction

We found a significant interaction between valence and component on gist memory, both with age as a three-tier category [*F*(2, 536) = 136.41, *p <* 0.001, adjusted *p <* 0.001, ηp^2^ = 0.34] and on a continuous scale [*F*(2, 1,350) = 4.37, *p =* 0.013, adjusted *p =* 0.048, ηp^2^ = 0.01]. Comparing object and background memory within negative, positive, and neutral scene type, memory for objects was better than that for backgrounds for both negative [*t*(273) = 17.06, *p <* 0.001, adjusted *p <* 0.001, *d =* 1.11] and positive scenes [*t*(273) = 19.41, *p <* 0.001, adjusted *p <* 0.*0*01, *d =* 1.22], although this object-background comparison was not significant for neutral scenes [*t*(273) = 1.59, *p =* 0.113, adjusted *p =* 0.113, *d =* 0.09].

In addition, both negative [*t*(273) = 9.33, *p <* 0.001, adjusted *p <* 0.001, *d =* 0.53] and positive objects [*t*(273) = 11.91, *p <* 0.001, adjusted *p <* 0.001, *d =* 0.62] were remembered better than neutral objects. In contrast, backgrounds associated with negative [*t*(273) = −8.24, *p <* 0.001, adjusted *p <* 0.001, *d =* −0.44] and positive objects [*t*(273) = −10.08, *p <* 0.001, adjusted *p <* 0.001, *d =* −0.47] were remembered worse than those associated with neutral objects, although memory did not differ for backgrounds presented on negative and positive scenes [*t*(273) = 0.94, *p =* 0.349, adjusted *p =* 0.349, *d =* 0.05]. Although memory for positive objects was numerically better than that for negative objects, this difference was not significant [*t*(273) = 1.66, *p =* 0.099, adjusted *p =* 0.099, *d =* 0.09].

In line with the valence by component interaction on memory, we also found a significant main effect of valence on memory trade-off effects treating age as both a three-tier category [*F*(2, 536) = 136.41, *p <* 0.001, adjusted *p <* 0.001, ηp^2^ = 0.34] and a continuous variable [*F*(2, 540) = 59.77, *p <* 0.001, adjusted *p <* 0.001, ηp^2^ = 0.18]. Follow-up *t*-tests indicated that negative [*t*(273) = 13.07, *p <* 0.001, adjusted *p <* 0.001, *d* = 0.96] and positive memory trade-off magnitudes [*t*(273) = 15.68, *p <* 0.001, adjusted *p <* 0.001, *d =* 1.09] were both greater than neutral one. Interestingly, the positive memory trade-off magnitude was numerically greater than the negative one [*t*(273) = 1.90, *p =* 0.059, adjusted *p =* 0.059, *d =* 0.13], although this effect was not significant.

#### 3.2.2 Age × valence interaction on gist memory

We found a significant interaction between age and valence on memory, both with age as a three-tier category [*F*(4, 536) = 4.84, *p =* 0.001, adjusted *p =* 0.004, ηp^2^ = 0.04] and on a continuous scale [*F*(2, 1,350) = 6.10, *p =* 0.002, adjusted *p =* 0.011, ηp^2^ = 0.01]. Although the three-way interaction between age, valence, and component was not significant with age being either a categorical [*F*(4, 536) = 0.53, *p* = 0.713, adjusted *p* = 0.823, ηp^2^ = 0.00] or continuous predictor [*F*(2, 1,350) = 0.33, *p* = 0.720, adjusted *p* = 0.806, ηp^2^ = 0.00], we conducted exploratory analyses to compare memory for objects and backgrounds separately as our research focus was not on memory collapsed across objects and backgrounds within each scene.

**Figure 3:**
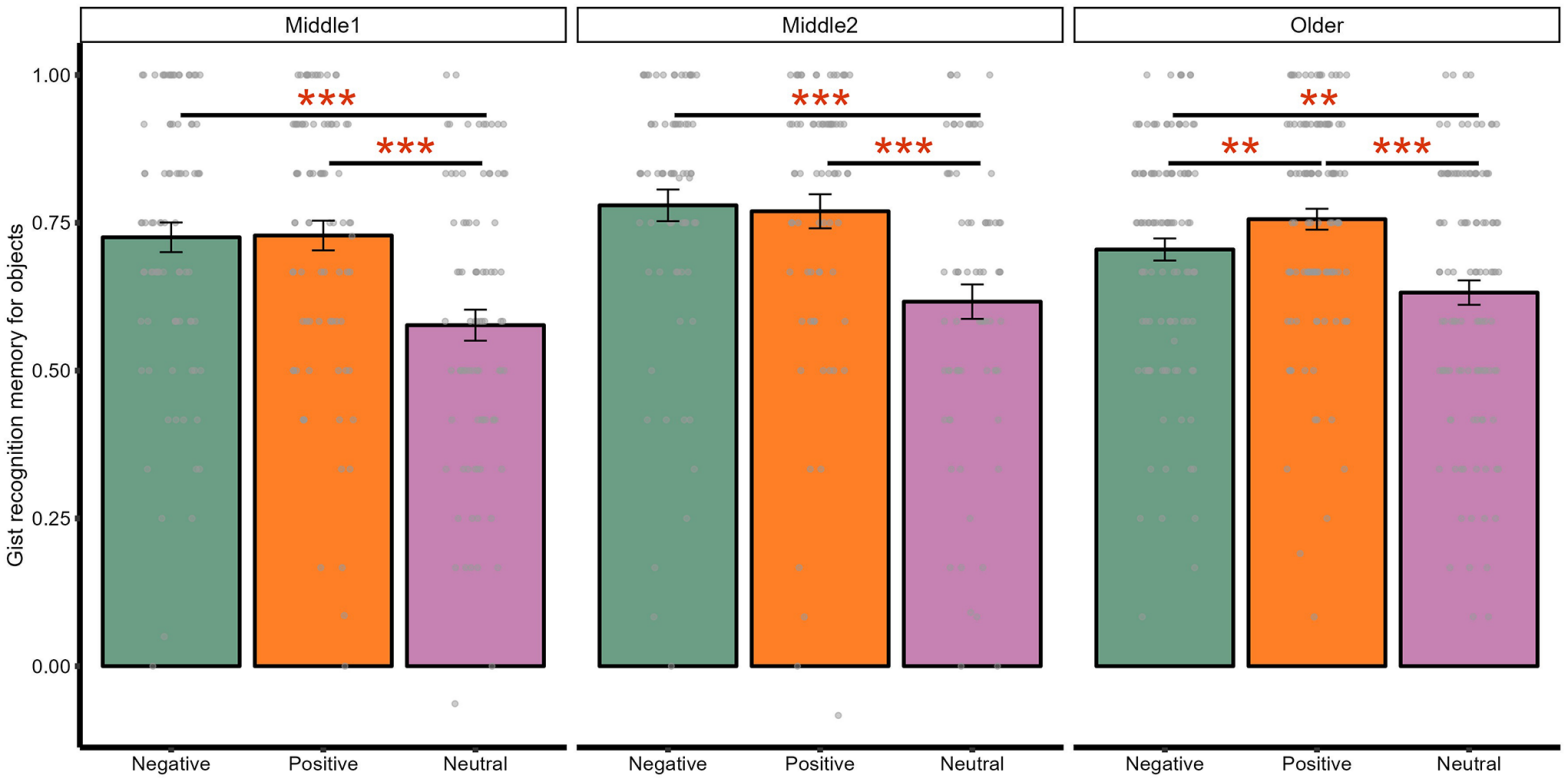
Interaction between age and valence on gist memory for objects. ***p < 0.001; **p < 0.01.

Comparing memory between negative and positive objects (Figure 3), older adults had significantly better memory for positive objects compared to negative ones [*t*(114) = 2.65, *p =* 0.009, adjusted *p =* 0.009, *d =* 0.26], while early and late middle-aged adults did not show significant differences (adjusted *ps* > 0.8, Supplementary Table S10). However, pairwise comparisons of memory between early middle-aged, late middle-aged, and older adults (adjusted *ps* > 0.9, Supplementary Table S9) and age-related correlations (adjusted *ps* > 0.6, Supplementary Table S14) were mostly not significant. Instead, all age groups showed similar patterns of better memory for both negative and positive objects compared to neutral objects, and worse memory for backgrounds presented on negative and positive scenes than neutral scenes (adjusted *ps* < 0.020, Supplementary Table S10).

#### 3.2.3 Age × valence interaction on memory trade-off effects

**Figure 4:**
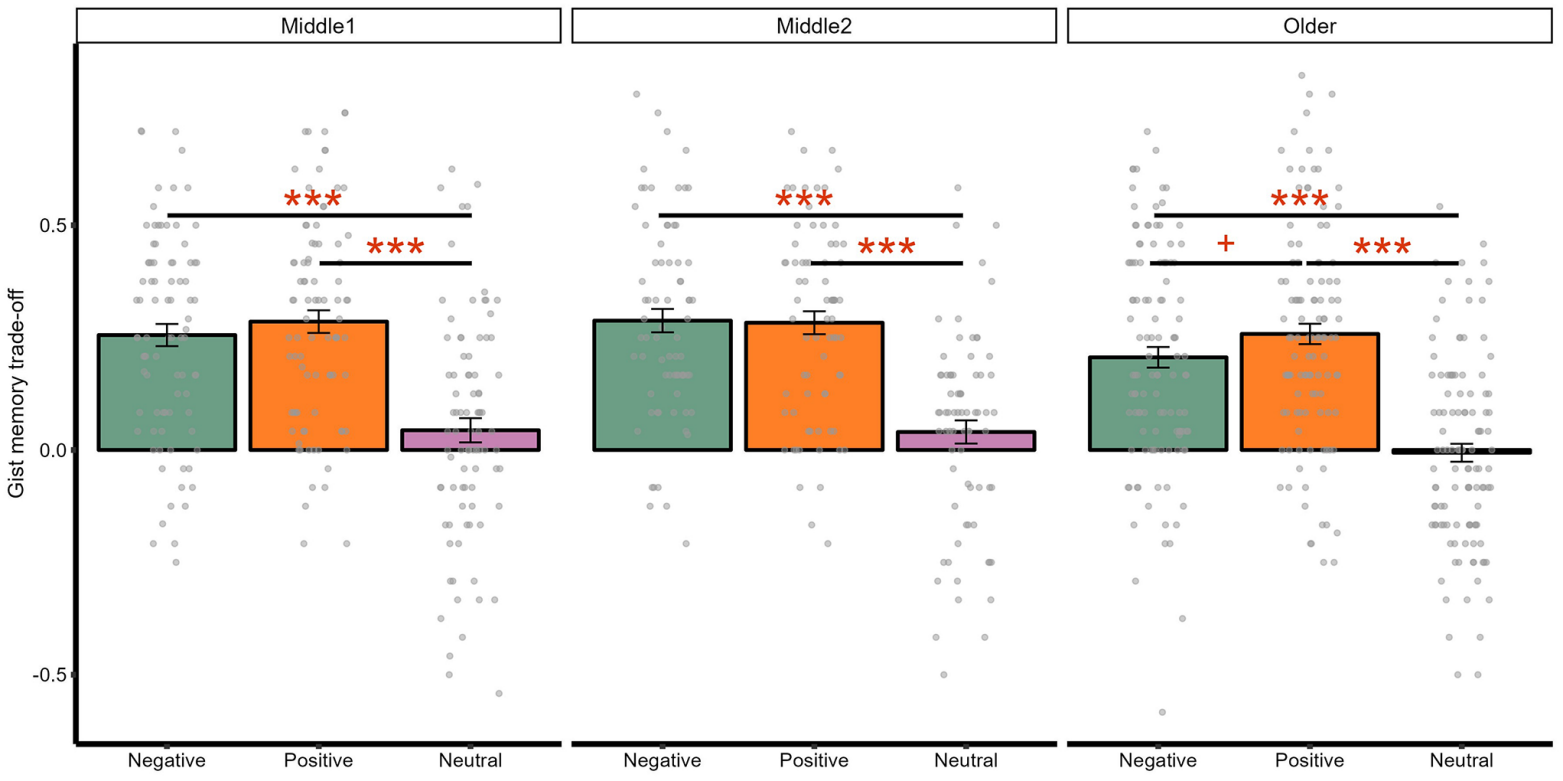
Interaction between age and valence on gist memory trade-off effects. ***p < 0.001; ^+^*p* < 0.10.

The interaction between age and valence was not significant, either with age being a categorical [*F*(4, 536) = 0.53, *p =* 0.713, adjusted *p =* 0.897, ηp^2^ = 0.00] or continuous predictor [*F*(2, 540) = 0.44, *p =* 0.647, adjusted *p =* 0.755, ηp^2^ = 0.00]. Exploratory analyses indicated that, when comparing positive and negative memory trade-off effects (Figure 4), only older adults had a numerically greater positive than negative memory trade-off magnitude [*t*(114) = 1.91, *p =* 0.059, adjusted *p =* 0.059, *d =* 0.21]. In contrast, neither early [*t*(85) = 1.14, *p =* 0.258, adjusted *p =* 0.258, *d =* 0.13] nor late middle-aged groups [*t*(72) = −0.17, *p =* 0.862, adjusted *p =* 0.862, *d =* −0.02] showed significant differences.

#### 3.2.4 The effects of valence and arousal ratings on gist memory

Negative objects were remembered significantly better when scenes containing the negative objects were rated as more arousing during encoding (*B* = 0.06, *p* = 0.003, adjusted *p* = 0.016, *R*^2^ = 0.03). Descriptively, memory trade-off effects for negative scenes were greater when negative scenes were rated as more negative (*B* = −0.06, *p* = 0.012, adjusted *p* = 0.059, *R*^2^ = 0.02) and more arousing (*B* = 0.05, *p* = 0.030, adjusted *p* = 0.089, *R*^2^ = 0.02). There were no other significant effects of valence and arousal ratings on recognition memory or memory trade-off effects (*ps* > 0.9, Supplementary Tables S15, S16).

### 3.3 The effects of delay conditions

Delay conditions did not yield significant main effects or interaction effects with age and/or scene valences on valence/arousal ratings, corrected recognition memory, or memory trade-off magnitude, either with age being a categorical or continuous predictor (*ps* > 0.9, Supplementary Tables S3, S6, S12).

## 4 Discussion

Our findings show that individuals interpret both positive and neutral information more positively as they approach older age, demonstrating the expected age-related positivity effect on valence ratings (i.e., positive interpretation bias). The age-related positivity effect also extends to memory. While none of the age groups exhibited a preference for remembering negative over positive information, older adults were more likely to remember positive than negative information. This was revealed as better memory for positive objects compared to negative objects on separate scenes, and descriptively speaking, greater positive than negative memory trade-off magnitude (i.e., the degree to which emotional objects were remembered better at the cost of their neutral peripheral backgrounds on the same scenes).

Consistent with prior research (Mather and Knight, 2005; Grühn and Scheibe, 2008; Gallo et al., 2009; Gilet et al., 2012; Schryer and Ross, 2012), we found that older age is correlated with perceiving positive events more positively. It is intriguing that older adults in our study also rated neutral scenes more positively, even though these stimuli have been previously normed as neutral in younger adults (Payne et al., 2008, 2012; Payne and Kensinger, 2011; Cunningham et al., 2014; Bennion et al., 2015, 2017; Denis et al., 2022; Sanders et al., n.d.). These results align with a previous study that this age-related positive interpretation bias also extends to neutral information (Mather and Knight, 2005). One possible mechanism underlying this interpretation bias is related to emotion regulation strategies that evolve with age. Specifically, compared to younger adults, older individuals tend to employ cognitive reappraisal to reframe their life experiences with positive interpretations (Liang et al., 2017; Nakagawa et al., 2017). This use of cognitive reappraisal and increased positive interpretation bias may contribute to a more positive overall outlook in older age, potentially promoting psychological resilience and satisfaction throughout the aging process (Höglund et al., 2020; Korkmaz and Güloğlu, 2021).

However, we failed to find a parallel association of increasing age and perceiving negative information less negatively. This is possibly because our negative stimuli consisted of highly negative content (e.g., snake, weapon, blood, vomit) that may have reached a functional ceiling with older participants, limiting the extent to which middle-aged adults could perceive them even more negatively. This potential ceiling effect might explain why universally negative information is not consistently perceived less negatively as individuals approach older age (Grühn and Scheibe, 2008; Streubel and Kunzmann, 2011; Gilet et al., 2012; Lee et al., 2023). In addition, our findings show that while middle-aged adults rated positive scenes as more arousing compared to neutral scenes, this pattern did not persist in older adults. Although age-related differences in arousal ratings did not reach significance, this may still point to a subtle shift in perceived arousal with age, consistent with previous findings on age-related decreases in perceived arousal to positive information (Keil and Freund, 2009; Gilet et al., 2012; Lee et al., 2023).

The finding that older adults remembered positive objects better than negative ones aligns with prior research that advancing age increases the positivity effect on memory (Reed et al., 2014). Notably, although this shift to a positivity memory bias is consistently found in studies where participants are instructed to passively view image or word stimuli (Charles et al., 2003; Leigland et al., 2004; Kensinger, 2008; Piguet et al., 2008; Spaniol et al., 2008), it is not observed in paradigms that impose informational processing constraints while viewing the stimuli (Kensinger et al., 2002; Denburg et al., 2003; Comblain et al., 2004; Grühn et al., 2005; Emery and Hess, 2008; Majerus and D’Argembeau, 2011; Leshikar et al., 2015; Eich and Castel, 2016). For example, when participants are explicitly instructed to remember stimuli (i.e., intentional encoding; Kensinger et al., 2002; Grühn et al., 2005; Emery and Hess, 2008; Majerus and D’Argembeau, 2011; Eich and Castel, 2016), or to make judgments about the stimuli during encoding, such as evaluating the complexity of visual images and determining whether verbal stimuli are self-referential (Comblain et al., 2004; Majerus and D’Argembeau, 2011), their ability to freely and naturally process emotional information may be hindered (Grühn et al., 2005; Gallo et al., 2009; Majerus and D’Argembeau, 2011). Therefore, the positivity effect may be attenuated when participants’ goals or focus are influenced by the experimental instruction (Carstensen and DeLiema, 2018; Sakaki et al., 2019).

Although we failed to find a linear association between older age and increasing positive memory bias, this is in accordance with prior research that age-related differences are only found when directly comparing older and younger, but not middle-aged adults (Fung et al., 2010; Reed et al., 2014). Critically, because most previous studies showing an age-related positivity effect focus on the comparisons between young and older adults (Leigland et al., 2004; Mather and Carstensen, 2005; Mikels et al., 2005; Kensinger, 2008; Piguet et al., 2008; Spaniol et al., 2008), it is not yet known whether this positivity effect already arises in middle age. The current study revealed that both early and late middle-aged adults do not show better memory for negative than positive information. This may suggest that the negativity bias and its influence on memory diminish in middle age, although a full transition to a positivity effect has not yet taken place. Another possibility for the null association between older age and increasing positive memory bias is that age-associated memory impairments counteract this positivity effect. Specifically, older adults tend to experience memory-related deficits due to declines in the structure and functionality of the hippocampus (Daselaar et al., 2006; de Flores et al., 2015). Although we focused on gist memory due to its resilience against forgetfulness, age-related memory deficits might still increase exponentially during the 12-h delay period (Davis et al., 2003; Titz and Verhaeghen, 2010; Matorina and Poppenk, 2021). Future research should incorporate retention intervals of varying lengths to explore the optimal delay condition during which positive aspects of memory are selectively consolidated more than accelerated forgetfulness impairs overall memory performance.

We replicated previous research findings on emotional memory trade-off effects, such that negative and positive objects were remembered better than both neutral objects on separate scenes and paired neutral backgrounds on the same scenes (Payne et al., 2008, 2012; Payne and Kensinger, 2011; Cunningham et al., 2014; Bennion et al., 2015, 2017; Denis et al., 2022; Sanders et al., n.d.). Interestingly, the typical pattern in younger adults, where neutral information in the background is less well-remembered when paired with a negative central object compared to a positive one (Denis et al., 2022; Sanders et al., n.d.), is not observed with middle-aged and older adults in the current study. Instead, in older age, the trade-off in emotional memory is more pronounced when the emotional object is positive as opposed to negative. This is potentially because emotionally positive events captures more attention than negative ones in older age (Noh and Isaacowitz, 2015; Erbey et al., 2020), thereby overshadowing the neutral and less salient aspects of the same event to a greater extent (Easterbrook, 1959; Reisberg and Heuer, 2004).

It is important to acknowledge that we did not explore how highly arousing, emotional information might have differential effects on memory bias from non-arousing, emotional information, especially because arousal and valence may influence memory processes through distinct mechanisms (Kensinger, 2004; Kensinger and Corkin, 2004). Prior research has shown that emotional memory induced by highly arousing information involves automatic processes and is well preserved with age (Kensinger, 2008), especially for negative stimuli (Mather and Knight, 2006; Leclerc and Kensinger, 2008). In contrast, emotional memory induced by non-arousing, negatively-valenced information tends to decline with age (Nashiro and Mather, 2011; Lee et al., 2023), as older adults may be utilizing cognitive control mechanisms via prefrontal cortex pathways to down-regulate their negative affect processing (Williams et al., 2006; St Jacques et al., 2009; Peña-Gómez et al., 2011). In line with prior research, our findings show that negative information is better remembered among middle-aged and older adults when it is perceived as arousing, but not when it is perceived as unpleasant. These results suggest that the age-related shift from negative to positive memory bias is most robust when comparing non-arousing or low-arousing, negative vs. positive information.

In our study, where most negative stimuli were rated as highly arousing relative to positive stimuli, it is not surprising that older age was not associated with worse memory for negative scenes, as they are likely still protected by the arousal pathway. Nevertheless, older adults still demonstrated superior memory for positive compared to negative information, which may suggest that age-related positivity effect observed in our study is likely driven primarily by valence-related emotional processing. Still, more studies should incorporate a broader spectrum of valence and arousal levels in their negative and positive stimuli, which is essential for exploring the nuanced impact of valence and arousal, both independently and interactively, on memory-related processes.

We failed to find sleep-dependent benefit for gist memory, which aligns with previous research indicating that meaning-based general properties in gist memory, relative to episodic-like details in specific memory, are less susceptible to forgetting over time and are therefore not as protected by sleep-related consolidation processes (Stickgold and Walker, 2013; Lutz et al., 2017; Matorina and Poppenk, 2021). Another plausible explanation could involve the fact that most participants in the sleep condition did not complete the encoding session immediately before their bedtime, whereas prior research demonstrates that sleep may only enhance memory consolidation when it occurs closely after learning (Solano et al., 2023). In addition, contrary to what we initially hypothesized based upon prior research, sleep did not magnify the emotional memory trade-off effect to result in better memory for emotional objects at the expense of associated neutral backgrounds, as has been seen before (Payne et al., 2008, 2012; Payne and Kensinger, 2011; Cunningham et al., 2014; Bennion et al., 2015, 2017). However, it is worth noting that most of these previous studies examined college-aged adults (Payne et al., 2008, 2012; Payne and Kensinger, 2011; Cunningham et al., 2014; Bennion et al., 2015, 2017), and the functional link between sleep and memory likely weakens with advancing age (Tucker et al., 2011; Scullin, 2013). Overall, this is in line with the existing literature that the prioritization of emotional memory during sleep is more of an exception than the norm (Prehn-Kristensen et al., 2009, 2013; Baran et al., 2012; Morgenthaler et al., 2014; Göder et al., 2015; Bolinger et al., 2018; Kurz et al., 2019; Davidson et al., 2021). Additionally, previous studies indicate that the proportion of rapid eye movement sleep and slow-wave sleep (Cairney et al., 2015) and the local coupling between sleep spindles and slow oscillations (Solano et al., 2021) both play crucial roles in memory consolidation. Future research with polysomnography-monitored sleep data is needed to investigate how different aspects of sleep architecture may facilitate age-related shifts in positive memory bias.

With a large online sample drawn from various U.S. communities, our findings provide better generalizability compared to laboratory studies with participants limited to college students or a single community. However, our sample may not fully represent the broader U.S. population as it consisted of healthy adults who were primarily White and had the technological literacy to participate using Prolific. It is also important to note that the attrition rate (50%) in our study was relatively high compared to in-person laboratory studies, as half of the eligible participants did not complete the follow-up experimental sessions. Although our attrition rate is still considered typical for longitudinal online studies, it may still impact the generalizability of our findings (Khadjesari et al., 2011; Rübsamen et al., 2017).

### 4.1 Conclusion

This is one of the first studies to examine emotional processing and delayed emotional memory performance in a sample of healthy middle age and older adults. Our findings support the age-related positivity effect in memory across a relatively long consolidation delay (approximately 12-h). Moreover, we provide well-powered evidence that while a shift toward positive *processing* emerges in middle age, the positivity bias in *memory* may not emerge until older adulthood. Efforts to further understand these age-related positivity effects, their mechanisms, boundary conditions, and impact on cognitive and emotional processing will be important if we are to better understand and promote mental well-being and cognitive functioning in aging adults.

## Data availability statement

The datasets presented in this study can be found in online repositories. The names of the repository/repositories and accession number(s) can be found below: https://doi.org/10.17605/OSF.IO/8Q2VW.

## Ethics statement

The studies involving humans were approved by The Institutional Review Board at the University of Notre Dame. The studies were conducted in accordance with the local legislation and institutional requirements. The participants provided their written informed consent to participate in this study.

## Author contributions

XN: Conceptualization, Data curation, Formal Analysis, Methodology, Project administration, Software, Validation, Visualization, Writing – original draft, Writing – review & editing. MU: Conceptualization, Methodology, Project administration, Writing – original draft, Writing – review & editing. KS: Data curation, Investigation, Methodology, Resources, Software, Supervision, Visualization, Writing – review & editing. EK: Funding acquisition, Investigation, Methodology, Resources, Supervision, Writing – review & editing. JP: Funding acquisition, Investigation, Methodology, Resources, Supervision, Writing – review & editing. DD: Data curation, Investigation, Methodology, Resources, Software, Visualization, Writing – review & editing.
