# Supplementary material for: Age-related positivity effect in emotional memory consolidation from middle age to late adulthood
Source: Front Behav Neurosci. 2024 Jan 24;18:1342589. doi: 10.3389/fnbeh.2024.1342589 (PMC10847278; doi:10.3389/fnbeh.2024.1342589)
Supplement: Supplementary file 1 [file Data_Sheet_1.docx]

Supplementary Material

# Methods

## Materials

### Sleep quality

The Pittsburgh Sleep Quality Index (PSQI) is a nine-item questionnaire that assesses various aspects of sleep habits and sleep quality during the past month (Buysse et al., 1989). Example items include “what time have you usually gone to bed at night?” and “how would you rate your sleep quality overall?”. The PSQI has seven subcomponents: sleep duration, sleep disturbance, sleep latency, sleep efficiency, subjective sleep quality, use of sleep medications, and daytime dysfunction. Scores on each subcomponent range from 0-3, yielding a composite PSQI score ranging from 0-21, with higher scores indicating greater sleep disturbance. The PSQI has acceptable to good reliability and validity (Buysse et al., 1989). The PSQI was completed during the initial screening survey.

### Psychomotor vigilance

We employed a brief 3-minute version of the Psychomotor Vigilance Test, known as the PVT-B, to detect changes in alertness associated with fatigue and sleep loss (Basner et al., 2011). Participants were informed that they would engage in a three-minute reaction time task, and they were instructed to keep their hands positioned above the spacebar throughout the task. Their task was to immediately press the spacebar when a red circle appeared on the screen, and not to press it when the screen was blank. The red circle's appearance was random, with interstimulus intervals ranging from one to four seconds. PVT scores were calculated by the average reaction time across all successfully completed trials. The PVT-B was administered before the scene rating task in session one and before the memory task in session two, and was programmed in jsPsych (de Leeuw, 2015) and hosted on Cognition.run (https://www.cognition.run).

### Sleepiness

The Stanford Sleepiness Scale (SSS) is a subjective measure of sleepiness (Hoddes et al., 1973). Respondents rate their current state using a 7-point scale, ranging from [1] “feeling active and vital; alert; wide awake” to [7] “almost in reverie; sleep onset soon; lost struggle to remain awake”. Thus, a higher score reflects before greater self-reported sleepiness. The SSS has demonstrated acceptable to good reliability and validity (Shahid et al., 2012) The SSS in the current study was completed at the beginning of session one, the PVT-B and scene viewing task, and again at the beginning of session two, before the PVT-B and memory task.

### Other measures

The following measures were not included in our analyses. During session 1, after the scene viewing task, participants completed the Morningness-Eveningness Questionnaire (Horne and Ostberg, 1976), the Mini-Mood and Anxiety Symptom Questionnaire (Casillas and Clark, 2000), and the Patient Depression Questionnaire (Kroenke et al., 2001), the Generalized Anxiety Disorder 7-item (Spitzer et al., 2006). During session 2, after the memory task, participants completed the Sleep Inertia Questionnaire (Kanady and Harvey, 2015), Beck's Depression Inventory (Beck et al., 1961), the Short Cognitive Emotion Regulation Questionnaire (Garnefski and Kraaij, 2006), and the Social Readjustment Rating Scale (Holmes and Rahe, 1967).

# Results

## Valence and arousal

Based on pairwise comparisons of negative, positive, and neutral scenes, positive scenes were rated as more positive than negative scenes (*t*(273)=48.20, *p<.001*, adjusted *p<.001*, *d=*4.75) and neutral scenes (*t*(273)=26.41, *p<.001*, adjusted *p<.001*, *d=*1.36), and rated as more arousing than neutral scenes (t(273)=3.90, *p<.001*, adjusted *p<.001*, *d=*0.19) but less arousing than negative scenes (*t*(273)=-21.52, *p<.001*, adjusted *p<.001*, *d=-*1.91). Negative scenes were rated as more negative (*t*(273)=-43.00, *p<.001*, adjusted *p<.001*, *d=*-3.74) and more arousing than and neutral scenes (*t*(273)=27.01, *p<.001*, adjusted *p<.001*, *d=*2.38).

## Specific memory

### Valence x component interaction

We found a significant interaction between valence and component on specific memory treating age as both a categorical (*F*(2,536) = 116.24, *p = .*000, adjusted *p = .*000, *ηp^2^* = 0.30) and continuous predictor (*F*(2,1350) = 5.80, *p = .*003, adjusted *p = .*016, *ηp^2^* = 0.01). Specific memory demonstrated similar effects as gist memory, such that emotional objects were remembered better than neutral objects, whereas backgrounds presented on emotional scenes were remembered worse than those on neutral scenes (adjusted *ps* < .001). However, there was a nonsignificant pattern that specific memory for positive objects was worse than that for negative objects (*t*(273) = -1.81, *p = .*072, adjusted *p = .*072, *d* = -0.09). Comparing object and background memory within each scene type, objects were remembered better than their paired backgrounds on negative (*t*(273) = 17.97, *p = .*000, adjusted *p = .*000, *d* = 1.17), positive (*t*(273) = 16.50, *p = .*000, adjusted *p = .*000, *d* = 1.02), as well as neutral scenes (*t*(273) = 2.26, *p = .*025, adjusted *p = .*025, *d* = 0.13).

### Age x valence interaction

We found a significant interaction between age and valence on specific memory when treating age as both a categorical (*F*(4,536) = 4.97, *p = .*001, adjusted *p = .*002, *ηp^2^* = 0.04) and continuous predictor (*F*(2,1350) = 4.61, *p = .*010, adjusted *p = .*038, *ηp^2^* = 0.01). However, the three-way interaction between age, valence, and component was not significant for either model (adjusted *ps* > .9). For specific memory, most pairwise comparisons between early middle-aged, late middle-aged, and older adults were not significant (adjusted *ps* > .9), with the exception that specific memory for neutral objects was better for older adults compared to early middle-aged adults (*t*(176) = 2.38, *p = .*018, adjusted *p = .*055, *d* = 0.34), which corresponded to a significant correlation between increasing age and better specific memory for neutral objects (*r* = 0.18, *p = .*003, adjusted *p = .*019).

Specific memory mostly followed the same pattern as gist memory when comparing emotional and neutral scene components, in the way that all age groups remembered emotional objects better than neutral objects (adjusted *ps* < .002). Interesting, although both early middle-aged and older adults had worse specific memory for backgrounds presented on emotional scenes compared to those presented on neutral scenes (adjusted *ps* < .001), late middle aged adults did not significantly remember backgrounds on negative (*t*(72) = -1.92, *p = .*059, adjusted *p = .*094, *d* = -0.21) or positive scenes (*t*(72) = -1.89, *p = .*063, adjusted *p = .*094, *d* = -0.18) worse than those on neutral scenes. In addition, while both early middle-aged and older adults did not have significantly different specific memory between positive and negative objects (adjusted *ps* > .5), late middle-aged adults significantly remembered negative objects better than positive ones (*t*(72) = 2.84, *p = .*006, adjusted *p = .*006, *d* = 0.30).

## Specific memory trade-off effects

### Main effect of valence

We found a significant main effect of valence on specific memory trade-off where age was a categorical (*F*(2,536) = 116.24, *p = .*000, adjusted *p = .*000, *ηp^2^* = 0.30) and continuous predictor (*F*(2,540) = 7.13, *p = .*001, adjusted *p = .*006, *ηp^2^* = 0.03). Follow-up *t*-tests indicated that negative (*t*(273) = 13.07, *p* = .000, adjusted *p* = 0.000, *d* = 0.96) and positive memory trade-off magnitudes (*t*(273) = 15.68, *p* = .000, adjusted *p* = 0.000, *d* = 1.09) were both greater than neutral ones. Additionally, negative memory trade-off magnitude was greater than positive one for specific memory (*t*(273) = 1.77, *p = .*078, adjusted *p = .*078, *d* = 0.12), although not significantly.

### Age x valence interaction

We failed to find a significant interaction between age and valence across either analysis (*ps* > .8). In fact, all age groups demonstrated a greater emotional than neutral memory trade-off magnitude for specific memory trade-off effects (adjusted *ps* <.001). For specific memory, the difference between negative and positive memory trade-off was not significant for early middle-aged (*t*(85) = 0.49, *p = .*622, adjusted *p = .*622, *d* = 0.06) and older adults (*t*(114) = 0.15, *p = .*881, adjusted *p = .*881, *d* = 0.02), but late middle-aged adults had greater negative than positive trade-off magnitude (*t*(72) = 2.79, *p = .*007, adjusted *p = .*007, *d* = 0.34).

No significant pairwise comparisons between early middle-aged, late middle-aged, and older adults were found for specific memory trade-off effects (adjusted *ps* > .9), and we failed to find any significant age-related correlations with any of the memory trade-off effects either (adjusted *ps* > .5).

# References

Basner, M., Mollicone, D., and Dinges, D. F. (2011). Validity and Sensitivity of a Brief Psychomotor Vigilance Test (PVT-B) to Total and Partial Sleep Deprivation. *Acta Astronaut* 69, 949–959. doi: 10.1016/j.actaastro.2011.07.015.

Beck, A. T., Ward, C. H., Mendelson, M., Mock, J., and Erbaugh, J. (1961). An inventory for measuring depression. *Arch Gen Psychiatry* 4, 561–571. doi: 10.1001/archpsyc.1961.01710120031004.

Buysse, D. J., Reynolds, C. F., Monk, T. H., Berman, S. R., and Kupfer, D. J. (1989). The Pittsburgh Sleep Quality Index: a new instrument for psychiatric practice and research. *Psychiatry Research* 28, 193–213. doi: 10.1016/0165-1781(89)90047-4.

Casillas, A., and Clark, L. A. (2000). The Mini Mood and Anxiety Symptom Questionnaire (Mini-MASQ). doi: 10.1037/e413792005-215.

de Leeuw, J. R. (2015). jsPsych: a JavaScript library for creating behavioral experiments in a Web browser. *Behav Res Methods* 47, 1–12. doi: 10.3758/s13428-014-0458-y.

Garnefski, N., and Kraaij, V. (2006). Cognitive emotion regulation questionnaire – development of a short 18-item version (CERQ-short). *Personality and Individual Differences* 41, 1045–1053. doi: 10.1016/j.paid.2006.04.010.

Hoddes, E., Zarcone, V., Smythe, H., Phillips, R., and Dement, W. C. (1973). Quantification of sleepiness: a new approach. *Psychophysiology* 10, 431–436. doi: 10.1111/j.1469-8986.1973.tb00801.x.

Holmes, T. H., and Rahe, R. H. (1967). The social readjustment rating scale. *Journal of Psychosomatic Research* 11, 213–218. doi: 10.1016/0022-3999(67)90010-4.

Horne, J. A., and Ostberg, O. (1976). A self-assessment questionnaire to determine morningness-eveningness in human circadian rhythms. *Int J Chronobiol* 4, 97–110.

Kanady, J. C., and Harvey, A. G. (2015). Development and Validation of the Sleep Inertia Questionnaire (SIQ) and Assessment of Sleep Inertia in Analogue and Clinical Depression. *Cognit Ther Res* 39, 601–612. doi: 10.1007/s10608-015-9686-4.

Kroenke, K., Spitzer, R. L., and Williams, J. B. (2001). The PHQ-9: validity of a brief depression severity measure. *J Gen Intern Med* 16, 606–613. doi: 10.1046/j.1525-1497.2001.016009606.x.

Shahid, A., Wilkinson, K., Marcu, S., and Shapiro, C. M. (2012). “Stanford Sleepiness Scale (SSS),” in *STOP, THAT and One Hundred Other Sleep Scales*, eds. A. Shahid, K. Wilkinson, S. Marcu, and C. M. Shapiro (New York, NY: Springer), 369–370. doi: 10.1007/978-1-4419-9893-4_91.

Spitzer, R. L., Kroenke, K., Williams, J. B. W., and Löwe, B. (2006). A brief measure for assessing generalized anxiety disorder: the GAD-7. *Arch Intern Med* 166, 1092–1097. doi: 10.1001/archinte.166.10.1092.

# Tables

## Table S1

*Demographic Characteristics of Early Middle-Aged, Late Middle-Aged, and Older Adults in the Nighttime Sleep and Daytime Wake Conditions*

|  | **Early Middle** | | **Late middle** | | | **Older** | | |
| --- | --- | --- | --- | --- | --- | --- | --- | --- |
|  | Sleep | Wake | | Sleep | Wake | | Sleep | Wake |
| Age (years) | 41.36 (3.57) | 40.73 (3.71) | | 53.94 (3.34) | 53.39 (3.51) | | 67.95 (5.34) | 67.34 (5.25) |
| Biological sex (%) |  |  | |  |  | |  |  |
| *Female* | 29% | 39% | | 66% | 50% | | 65% | 59% |
| *Male* | 71% | 61% | | 34% | 50% | | 35% | 41% |
| *Intersex* | 0% | 0% | | 0% | 0% | | 0% | 0% |
| Ethnicity (%) |  |  | |  |  | |  |  |
| *Spanish* | 0% | 0% | | 0% | 3% | | 0% | 0% |
| *Hispanic* | 0% | 0% | | 3% | 5% | | 0% | 0% |
| *Latino* | 4% | 5% | | 3% | 0% | | 0% | 0% |
| *Hispanic & Latino* | 2% | 2% | | 0% | 0% | | 0% | 0% |
| *None of these* | 93% | 93% | | 94% | 92% | | 100% | 100% |
| Race (%) |  |  | |  |  | |  |  |
| *White* | 76% | 85% | | 86% | 79% | | 93% | 97% |
| *Black or  African American* | 7% | 5% | | 9% | 11% | | 5% | 3% |
| *American Indian or  Alaska Native* | 2% | 2% | | 0% | 0% | | 0% | 0% |
| *Asian* | 13% | 7% | | 6% | 11% | | 2% | 0% |
| *None of these* | 2% | 0% | | 0% | 0% | | 0% | 0% |
| Household income | 78742 (58926) | 58956 (42863) | | 84871 (62068) | 82375 (50074) | | 64162 (48400) | 77015 (41640) |
| *Note.* Mean (standard deviation) are displayed for age and household income. Percentage is displayed for biological sex, ethnicity, and race. Early middle, 35-47 years old. Late middle, 48-59 years old. Older, at least 60 years old. | | | | | | | | |

## Table S2

*Descriptive Statistics of Valence and Arousal Ratings During Encoding for Early Middle-Aged, Late Middle-Aged, Older Adults, and the Total Sample*

|  |  | **Valence** | | **Arousal** | |
| --- | --- | --- | --- | --- | --- |
|  |  | *M* | *SD* | *M* | *SD* |
| Negative scenes | Early middle | 2.57 | 0.72 | 5.13 | 0.77 |
|  | Late middle | 2.42 | 0.51 | 5.37 | 0.57 |
|  | Older | 2.51 | 0.57 | 5.31 | 0.66 |
|  | **Total** | **2.51** | **0.61** | **5.27** | **0.68** |
| Positive scenes | Early middle | 5.37 | 0.72 | 3.90 | 0.85 |
|  | Late middle | 5.67 | 0.66 | 3.93 | 0.83 |
|  | Older | 5.61 | 0.63 | 3.66 | 0.85 |
|  | **Total** | **5.55** | **0.68** | **3.81** | **0.85** |
| Neutral scenes | Early middle | 4.57 | 0.53 | 3.68 | 0.69 |
|  | Late middle | 4.79 | 0.66 | 3.71 | 0.72 |
|  | Older | 4.75 | 0.52 | 3.61 | 0.64 |
|  | **Total** | **4.70** | **0.57** | **3.66** | **0.67** |
| *Note.* Early middle, 35-47 years old. Late middle, 48-59 years old. Older, at least 60 years old. | | | | | |

## Table S3

*Analysis of Variance Test Results for Valence and Arousal Ratings During Encoding*

|  | **Age as a categorical predictor** | | | |  | **Age as a continuous predictor** | | | |
| --- | --- | --- | --- | --- | --- | --- | --- | --- | --- |
|  | *F* | *p* | *p.*adj | *ηp2* |  | *F* | *p* | *p.*adj | *ηp2* |
| ***Valence Ratings*** |  | | | |  |  | | | |
| Age | F(2, 268)=2.78 | .064 | .149 | 0.02 |  | F(2, 268)=1.75 | .175 | .306 | 0.01 |
| Valence | F(2, 536)=1906.28 | .000 | .000 | 0.88 |  | F(2, 536)=480.86 | .000 | .000 | 0.64 |
| Delay | F(1, 268)=1.78 | .184 | .322 | 0.01 |  | F(1, 268)=0.16 | .694 | .712 | 0.00 |
| Age x Valence | F(4, 536)=3.38 | .010 | .035 | 0.03 |  | F(4, 536)=2.79 | .026 | .091 | 0.02 |
| Age x Delay | F(2, 268)=0.85 | .429 | .601 | 0.01 |  | F(2, 268)=0.34 | .712 | .712 | 0.00 |
| Valence x Delay | F(2, 536)=0.41 | .663 | .663 | 0.00 |  | F(2, 536)=2.39 | .093 | .217 | 0.01 |
| Age x Valence x Delay | F(4, 536)=0.79 | .535 | .624 | 0.01 |  | F(4, 536)=1.12 | .345 | .483 | 0.01 |
| ***Arousal Ratings*** |  | | | |  |  | | | |
| Age | F(1, 270)=3.92 | .049 | .120 | 0.01 |  | F(1, 270)=0.51 | .475 | .967 | 0.00 |
| Valence | F(2, 540)=59.77 | .000 | .000 | 0.18 |  | F(2, 540)=9.90 | .000 | .000 | 0.04 |
| Delay | F(1, 270)=1.25 | .265 | .464 | 0.00 |  | F(1, 270)=0.23 | .631 | .967 | 0.00 |
| Age x Valence | F(2, 540)=2.98 | .052 | .120 | 0.01 |  | F(2, 540)=3.91 | .021 | .072 | 0.01 |
| Age x Delay | F(1, 270)=0.78 | .379 | .517 | 0.00 |  | F(1, 270)=0.15 | .703 | .967 | 0.00 |
| Valence x Delay | F(2, 540)=0.66 | .517 | .517 | 0.00 |  | F(2, 540)=0.03 | .967 | .967 | 0.00 |
| Age x Valence x Delay | F(2, 540)=0.71 | .491 | .517 | 0.00 |  | F(2, 540)=0.15 | .861 | .967 | 0.00 |
| *Note.* Mixed-model ANOVA with a 3 (Age: Early Middle Age, Late Middle Age, Older Adulthood) x 3 (Scene Valence: Negative, Neutral, Positive) x 2 (Delay: Sleep, Wake) design, and linear mixed-effects ANOVA treating age as a continuous variable were performed for valence and arousal ratings separately. Early middle, 35-47 years old. Late middle, 48-59 years old. Older, at least 60 years old. *p*.adj, *p*-values adjusted for the false discovery rate (FDR). *ηp^2^*, effect size measured by partial eta-squared. | | | | | | | | | |

## Table S4

*Pairwise T-Tests of Age and Scene Valence for Valence and Arousal Ratings During Encoding*

|  |  |  | **Valence** | | | |  | **Arousal** | | | |
| --- | --- | --- | --- | --- | --- | --- | --- | --- | --- | --- | --- |
|  |  |  | *t* | *p* | *p*.adj | *d* |  | *t* | *p* | *p*.adj | *d* |
| *Comparing early middle, late middle, and older age* | | | |  |  |  |  |  |  |  |  |
| Negative scenes | Middle1 | Middle2 | t(153)=1.48 | .141 | .375 | 0.23 |  | t(154)=-2.23 | .027 | .082 | -0.35 |
|  | Middle1 | Older | t(158)=0.56 | .577 | .577 | 0.08 |  | t(166)=-1.74 | .083 | .125 | -0.25 |
|  | Middle2 | Older | t(165)=-1.15 | .250 | .375 | -0.17 |  | t(169)=0.63 | .528 | .528 | 0.09 |
| Positive scenes | Middle1 | Middle2 | t(156)=-2.77 | .006 | .019 | -0.44 |  | t(154)=-0.23 | .821 | .821 | -0.04 |
|  | Middle1 | Older | t(170)=-2.41 | .017 | .026 | -0.35 |  | t(183)=2.02 | .044 | .067 | 0.29 |
|  | Middle2 | Older | t(149)=0.70 | .486 | .486 | 0.10 |  | t(156)=2.20 | .029 | .067 | 0.33 |
| Neutral scenes | Middle1 | Middle2 | t(138)=-2.21 | .029 | .044 | -0.35 |  | t(151)=-0.23 | .817 | .817 | -0.04 |
|  | Middle1 | Older | t(181)=-2.35 | .020 | .044 | -0.34 |  | t(176)=0.71 | .479 | .718 | 0.10 |
|  | Middle2 | Older | t(127)=0.39 | .696 | .696 | 0.06 |  | t(140)=0.91 | .365 | .718 | 0.14 |
| *Comparing negative, positive, and neutral scenes* | | | |  |  |  |  |  |  |  |  |
| Early middle | Negative | Positive | t(85)=-21.81 | .000 | .000 | -3.92 |  | t(85)=9.25 | .000 | .000 | 1.52 |
|  | Negative | Neutral | t(85)=-20.99 | .000 | .000 | -3.18 |  | t(85)=12.07 | .000 | .000 | 1.99 |
|  | Positive | Neutral | t(85)=12.23 | .000 | .000 | 1.26 |  | t(85)=3.27 | .002 | .002 | 0.28 |
| Late middle | Negative | Positive | t(72)=-31.48 | .000 | .000 | -5.52 |  | t(72)=11.69 | .000 | .000 | 2.03 |
|  | Negative | Neutral | t(72)=-24.76 | .000 | .000 | -4.00 |  | t(72)=14.09 | .000 | .000 | 2.57 |
|  | Positive | Neutral | t(72)=15.31 | .000 | .000 | 1.35 |  | t(72)=2.78 | .007 | .007 | 0.29 |
| Older | Negative | Positive | t(114)=-33.28 | .000 | .000 | -5.14 |  | t(114)=16.74 | .000 | .000 | 2.18 |
|  | Negative | Neutral | t(114)=-29.49 | .000 | .000 | -4.11 |  | t(114)=21.21 | .000 | .000 | 2.62 |
|  | Positive | Neutral | t(114)=18.63 | .000 | .000 | 1.48 |  | t(114)=0.81 | .421 | .421 | 0.06 |
| *Note.* Early middle, 35-47 years old. Late middle, 48-59 years old. Older, at least 60 years old. *p*.adj, *p*-values adjusted for the false discovery rate (FDR). *d*, effect size measured by Cohen’s *d*. | | | | | | | | | | | |

## Table S5

*Descriptive Statistics of Gist and Specific Memory for Early Middle-Aged, Late Middle-Aged, Older Adults, and the Total Sample*

|  |  | **Gist memory** | | **Specific memory** | |
| --- | --- | --- | --- | --- | --- |
|  |  | *M* | *SD* | *M* | *SD* |
| Negative object | Early middle | 0.73 | 0.23 | 0.53 | 0.28 |
|  | Late middle | 0.78 | 0.23 | 0.58 | 0.27 |
|  | Older | 0.70 | 0.20 | 0.53 | 0.28 |
|  | **Total** | **0.73** | **0.22** | **0.54** | **0.28** |
| Negative background | Early middle | 0.47 | 0.22 | 0.26 | 0.21 |
|  | Late middle | 0.49 | 0.21 | 0.27 | 0.19 |
|  | Older | 0.50 | 0.22 | 0.26 | 0.19 |
|  | **Total** | **0.49** | **0.22** | **0.26** | **0.20** |
| Positive object | Early middle | 0.73 | 0.23 | 0.51 | 0.27 |
|  | Late middle | 0.77 | 0.25 | 0.49 | 0.29 |
|  | Older | 0.76 | 0.19 | 0.54 | 0.27 |
|  | **Total** | **0.75** | **0.22** | **0.52** | **0.27** |
| Positive background | Early middle | 0.44 | 0.24 | 0.25 | 0.23 |
|  | Late middle | 0.49 | 0.24 | 0.28 | 0.22 |
|  | Older | 0.50 | 0.22 | 0.28 | 0.21 |
|  | **Total** | **0.48** | **0.23** | **0.27** | **0.22** |
| Neutral object | Early middle | 0.58 | 0.24 | 0.34 | 0.26 |
|  | Late middle | 0.62 | 0.25 | 0.32 | 0.24 |
|  | Older | 0.63 | 0.22 | 0.38 | 0.24 |
|  | **Total** | **0.61** | **0.24** | **0.35** | **0.25** |
| Neutral background | Early middle | 0.53 | 0.26 | 0.34 | 0.25 |
|  | Late middle | 0.58 | 0.25 | 0.36 | 0.25 |
|  | Older | 0.64 | 0.22 | 0.42 | 0.23 |
|  | **Total** | **0.59** | **0.25** | **0.38** | **0.24** |
| *Note.* Early middle, 35-47 years old. Late middle, 48-59 years old. Older, at least 60 years old. | | | | | |

## Table S6

*Analysis of Variance Test Results for Gist Recognition Memory*

|  | **Age as a categorical predictor** | | | |  | **Age as a continuous predictor** | | | |
| --- | --- | --- | --- | --- | --- | --- | --- | --- | --- |
|  | *F* | *p* | *p.*adj | *ηp2* |  | *F* | *p* | *p.*adj | *ηp2* |
| Age | F(2, 268)=1.63 | .198 | .297 | 0.01 |  | F(1, 270)=3.75 | .054 | .135 | 0.01 |
| Valence | F(2, 536)=2.80 | .062 | .225 | 0.01 |  | F(2, 1350)=6.71 | .001 | .009 | 0.01 |
| Component | F(1, 268)=301.96 | .000 | .000 | 0.53 |  | F(1, 1350)=49.48 | .000 | .000 | 0.04 |
| Delay | F(1, 268)=2.47 | .117 | .225 | 0.01 |  | F(1, 270)=0.65 | .420 | .701 | 0.00 |
| Age x Valence | F(4, 536)=4.84 | .001 | .004 | 0.04 |  | F(2, 1350)=6.10 | .002 | .011 | 0.01 |
| Age x Component | F(2, 268)=2.49 | .085 | .225 | 0.02 |  | F(1, 1350)=4.31 | .038 | .114 | 0.00 |
| Valence x Component | F(2, 536)=136.41 | .000 | .000 | 0.34 |  | F(2, 1350)=4.37 | .013 | .048 | 0.01 |
| Age x Delay | F(2, 268)=1.04 | .354 | .443 | 0.01 |  | F(1, 270)=1.36 | .245 | .524 | 0.01 |
| Valence x Delay | F(2, 536)=1.38 | .253 | .345 | 0.01 |  | F(2, 1350)=0.39 | .678 | .806 | 0.00 |
| Component x Delay | F(1, 268)=1.84 | .176 | .293 | 0.01 |  | F(1, 1350)=0.18 | .671 | .806 | 0.00 |
| Age x Valence x Component | F(4, 536)=0.53 | .713 | .823 | 0.00 |  | F(2, 1350)=0.33 | .720 | .806 | 0.00 |
| Age x Valence x Delay | F(4, 536)=1.90 | .109 | .225 | 0.01 |  | F(2, 1350)=0.23 | .795 | .806 | 0.00 |
| Age x Component x Delay | F(2, 268)=0.11 | .897 | .897 | 0.00 |  | F(1, 1350)=0.75 | .387 | .701 | 0.00 |
| Valence x Component x Delay | F(2, 536)=2.13 | .120 | .225 | 0.01 |  | F(2, 1350)=0.22 | .806 | .806 | 0.00 |
| Age x Valence x Component x Delay | F(4, 536)=0.30 | .880 | .897 | 0.00 |  | F(2, 1350)=0.23 | .795 | .806 | 0.00 |
| *Note.* Mixed-model ANOVA with a 3 (Age: Early Middle Age, Late Middle Age, Older Adulthood) x 3 (Scene Valence: Negative, Neutral, Positive) x 2 (Component: Object, Background) x 2 (Delay: Sleep, Wake) design, and linear mixed-effects ANOVA treating age as a continuous variable was performed for gist memory. Early middle, 35-47 years old. Late middle, 48-59 years old. Older, at least 60 years old. *p*.adj, *p*-values adjusted for the false discovery rate (FDR). *ηp^2^*, effect size measured by partial eta-squared. | | | | | | | | | |

## Table S7

*Analysis of Variance Test Results for Specific Recognition Memory*

|  | **Age as a categorical predictor** | | | |  | **Age as a continuous predictor** | | | |
| --- | --- | --- | --- | --- | --- | --- | --- | --- | --- |
|  | *F* | *p* | *p.*adj | *ηp2* |  | *F* | *p* | *p.*adj | *ηp2* |
| Age | F(2, 268)=0.62 | .540 | .810 | 0.01 |  | F(1, 270)=3.10 | .079 | .238 | 0.01 |
| Valence | F(2, 536)=15.89 | .000 | .000 | 0.06 |  | F(2, 1350)=7.38 | .001 | .005 | 0.01 |
| Component | F(1, 268)=285.20 | .000 | .000 | 0.52 |  | F(1, 1350)=11.93 | .001 | .005 | 0.01 |
| Delay | F(1, 268)=5.02 | .026 | .078 | 0.02 |  | F(1, 270)=0.07 | .792 | .884 | 0.00 |
| Age x Valence | F(4, 536)=4.98 | .001 | .002 | 0.04 |  | F(2, 1350)=4.61 | .010 | .038 | 0.01 |
| Age x Component | F(2, 268)=0.20 | .822 | .881 | 0.00 |  | F(1, 1350)=2.52 | .113 | .282 | 0.00 |
| Valence x Component | F(2, 536)=116.24 | .000 | .000 | 0.30 |  | F(2, 1350)=5.80 | .003 | .016 | 0.01 |
| Age x Delay | F(2, 268)=0.50 | .609 | .823 | 0.00 |  | F(1, 270)=0.05 | .817 | .884 | 0.00 |
| Valence x Delay | F(2, 536)=1.22 | .297 | .557 | 0.01 |  | F(2, 1350)=1.09 | .336 | .720 | 0.00 |
| Component x Delay | F(1, 268)=3.58 | .060 | .150 | 0.01 |  | F(1, 1350)=0.08 | .780 | .884 | 0.00 |
| Age x Valence x Component | F(4, 536)=1.45 | .216 | .463 | 0.01 |  | F(2, 1350)=0.10 | .906 | .906 | 0.00 |
| Age x Valence x Delay | F(4, 536)=0.46 | .764 | .881 | 0.00 |  | F(2, 1350)=0.81 | .446 | .743 | 0.00 |
| Age x Component x Delay | F(2, 268)=0.42 | .658 | .823 | 0.00 |  | F(1, 1350)=0.72 | .395 | .740 | 0.00 |
| Valence x Component x Delay | F(2, 536)=0.98 | .377 | .628 | 0.00 |  | F(2, 1350)=0.19 | .825 | .884 | 0.00 |
| Age x Valence x Component x Delay | F(4, 536)=0.26 | .904 | .904 | 0.00 |  | F(2, 1350)=0.29 | .752 | .884 | 0.00 |
| *Note.* Mixed-model ANOVA with a 3 (Age: Early Middle Age, Late Middle Age, Older Adulthood) x 3 (Scene Valence: Negative, Neutral, Positive) x 2 (Component: Object, Background) x 2 (Delay: Sleep, Wake) design, and linear mixed-effects ANOVA treating age as a continuous variable was performed for specific memory. Early middle, 35-47 years old. Late middle, 48-59 years old. Older, at least 60 years old. *p*.adj, *p*-values adjusted for the false discovery rate (FDR). *ηp^2^*, effect size measured by partial eta-squared. | | | | | | | | | |

## Table S8

*Pairwise T-Tests of Component and Scene Valence on Gist and Specific Memory*

|  |  |  | **Gist Memory** | | | |  | **Specific Memory** | | | |
| --- | --- | --- | --- | --- | --- | --- | --- | --- | --- | --- | --- |
|  |  |  | *t* | *p* | *p*.adj | *d* |  | *t* | *p* | *p*.adj | *d* |
| *Comparing objects and backgrounds* | | | |  |  |  |  |  |  |  |  |
| Negative scenes | Object | Background | t(273)=17.06 | .000 | .000 | 1.11 |  | t(273)=17.97 | .000 | .000 | 1.17 |
| Positive scenes | Object | Background | t(273)=19.41 | .000 | .000 | 1.22 |  | t(273)=16.50 | .000 | .000 | 1.02 |
| Neutral scenes | Object | Background | t(273)=1.59 | .113 | .113 | 0.09 |  | t(273)=2.26 | .025 | .025 | 0.13 |
| *Comparing negative, positive, and neutral scenes* | | | |  |  |  |  |  |  |  |  |
| Object | Negative | Positive | t(273)=-1.66 | .099 | .099 | -0.09 |  | t(273)=1.81 | .072 | .072 | 0.09 |
|  | Negative | Neutral | t(273)=9.33 | .000 | .000 | 0.53 |  | t(273)=11.92 | .000 | .000 | 0.63 |
|  | Positive | Neutral | t(273)=11.91 | .000 | .000 | 0.62 |  | t(273)=10.83 | .000 | .000 | 0.54 |
| Background | Negative | Positive | t(273)=0.94 | .349 | .349 | 0.05 |  | t(273)=-0.57 | .571 | .571 | -0.03 |
|  | Negative | Neutral | t(273)=-8.24 | .000 | .000 | -0.44 |  | t(273)=-7.02 | .000 | .000 | -0.38 |
|  | Positive | Neutral | t(273)=-10.08 | .000 | .000 | -0.47 |  | t(273)=-6.93 | .000 | .000 | -0.34 |
| *Note.* *p*.adj, *p*-values adjusted for the false discovery rate (FDR). *d*, effect size measured by Cohen’s *d*. | | | | | | | | | | | |

## Table S9

*Pairwise T-Tests of Age for Gist and Specific Memory Within Each Scene Component*

|  |  |  | **Gist memory** | | | |  | **Specific memory** | | | |
| --- | --- | --- | --- | --- | --- | --- | --- | --- | --- | --- | --- |
|  |  |  | *t* | *p* | *p*.adj | *d* |  | *t* | *p* | *p*.adj | *d* |
| *Negative scenes* | |  |  |  |  |  |  |  |  |  |  |
| Object | Middle1 | Middle2 | t(154)=-1.47 | .143 | .214 | -0.23 |  | t(153)=-1.05 | .296 | .444 | -0.17 |
|  | Middle1 | Older | t(167)=0.66 | .511 | .511 | 0.09 |  | t(183)=0.05 | .959 | .959 | 0.01 |
|  | Middle2 | Older | t(139)=2.29 | .024 | .071 | 0.35 |  | t(154)=1.17 | .246 | .444 | 0.17 |
| Background | Middle1 | Middle2 | t(154)=-0.64 | .520 | .780 | -0.10 |  | t(155)=-0.37 | .709 | .968 | -0.06 |
|  | Middle1 | Older | t(186)=-0.92 | .356 | .780 | -0.13 |  | t(177)=0.04 | .968 | .968 | 0.01 |
|  | Middle2 | Older | t(159)=-0.21 | .831 | .831 | -0.03 |  | t(154)=0.45 | .653 | .968 | 0.07 |
| *Positive scenes* | |  |  |  |  |  |  |  |  |  |  |
| Object | Middle1 | Middle2 | t(150)=-1.07 | .286 | .556 | -0.17 |  | t(149)=0.34 | .736 | .736 | 0.05 |
|  | Middle1 | Older | t(161)=-0.90 | .371 | .556 | -0.13 |  | t(183)=-0.88 | .378 | .567 | -0.13 |
|  | Middle2 | Older | t(125)=0.40 | .693 | .693 | 0.06 |  | t(145)=-1.16 | .248 | .567 | -0.18 |
| Background | Middle1 | Middle2 | t(152)=-1.15 | .251 | .376 | -0.18 |  | t(155)=-0.75 | .455 | .682 | -0.12 |
|  | Middle1 | Older | t(175)=-1.69 | .093 | .279 | -0.24 |  | t(174)=-0.80 | .426 | .682 | -0.11 |
|  | Middle2 | Older | t(143)=-0.33 | .739 | .739 | -0.05 |  | t(149)=0.04 | .966 | .966 | 0.01 |
| *Neutral scenes* | |  |  |  |  |  |  |  |  |  |  |
| Object | Middle1 | Middle2 | t(152)=-1.01 | .312 | .468 | -0.16 |  | t(152)=-0.64 | .520 | .520 | -0.10 |
|  | Middle1 | Older | t(173)=-1.65 | .101 | .303 | -0.24 |  | t(176)=-2.38 | .018 | .055 | -0.34 |
|  | Middle2 | Older | t(140)=-0.43 | .668 | .668 | -0.07 |  | t(144)=-1.55 | .124 | .186 | -0.23 |
| Background | Middle1 | Middle2 | t(154)=-1.06 | .291 | .291 | -0.17 |  | t(157)=0.39 | .698 | .698 | 0.06 |
|  | Middle1 | Older | t(161)=-3.02 | .003 | .009 | -0.44 |  | t(171)=-1.12 | .263 | .394 | -0.16 |
|  | Middle2 | Older | t(135)=-1.71 | .089 | .133 | -0.26 |  | t(153)=-1.58 | .116 | .348 | -0.24 |
| *Note.* Early middle, 35-47 years old. Late middle, 48-59 years old. Older, at least 60 years old. *p*.adj, *p*-values adjusted for the false discovery rate (FDR). *d*, effect size measured by Cohen’s *d*. | | | | | | | | | | | |

## Table S10

*Pairwise T-Tests of Scene Valence for Gist and Specific Memory Within Each Age Group*

|  |  |  | **Gist memory** | | | |  | **Specific memory** | | | |
| --- | --- | --- | --- | --- | --- | --- | --- | --- | --- | --- | --- |
|  |  |  | *t* | *p* | *p*.adj | *d* |  | *t* | *p* | *p*.adj | *d* |
| *Early middle age* | |  |  |  |  |  |  |  |  |  |  |
| Object | Negative | Positive | t(85)=-0.17 | .868 | .868 | -0.01 |  | t(85)=1.10 | .274 | .274 | 0.08 |
|  | Negative | Neutral | t(85)=7.12 | .000 | .000 | 0.62 |  | t(85)=8.93 | .000 | .000 | 0.74 |
|  | Positive | Neutral | t(85)=7.60 | .000 | .000 | 0.64 |  | t(85)=7.88 | .000 | .000 | 0.67 |
| Background | Negative | Positive | t(85)=1.41 | .162 | .162 | 0.12 |  | t(85)=0.52 | .606 | .606 | 0.04 |
|  | Negative | Neutral | t(85)=-2.78 | .007 | .010 | -0.26 |  | t(85)=-3.64 | .000 | .001 | -0.31 |
|  | Positive | Neutral | t(85)=-4.33 | .000 | .000 | -0.36 |  | t(85)=-3.94 | .000 | .001 | -0.34 |
| *Late middle age* | |  |  |  |  |  |  |  |  |  |  |
| Object | Negative | Positive | t(72)=0.41 | .681 | .681 | 0.04 |  | t(72)=2.84 | .006 | .006 | 0.30 |
|  | Negative | Neutral | t(72)=6.25 | .000 | .000 | 0.68 |  | t(72)=7.52 | .000 | .000 | 0.82 |
|  | Positive | Neutral | t(72)=6.95 | .000 | .000 | 0.62 |  | t(72)=5.34 | .000 | .000 | 0.49 |
| Background | Negative | Positive | t(72)=0.26 | .798 | .798 | 0.02 |  | t(72)=-0.29 | .775 | .775 | -0.03 |
|  | Negative | Neutral | t(72)=-3.53 | .001 | .001 | -0.36 |  | t(72)=-1.92 | .059 | .094 | -0.21 |
|  | Positive | Neutral | t(72)=-4.13 | .000 | .000 | -0.37 |  | t(72)=-1.89 | .063 | .094 | -0.18 |
| *Older age* | |  |  |  |  |  |  |  |  |  |  |
| Object | Negative | Positive | t(114)=-2.65 | .009 | .009 | -0.26 |  | t(114)=-0.64 | .521 | .521 | -0.05 |
|  | Negative | Neutral | t(114)=3.63 | .000 | .001 | 0.35 |  | t(114)=5.20 | .000 | .000 | 0.43 |
|  | Positive | Neutral | t(114)=6.42 | .000 | .000 | 0.60 |  | t(114)=5.84 | .000 | .000 | 0.49 |
| Background | Negative | Positive | t(114)=0.05 | .963 | .963 | 0.00 |  | t(114)=-1.07 | .289 | .289 | -0.08 |
|  | Negative | Neutral | t(114)=-7.89 | .000 | .000 | -0.64 |  | t(114)=-6.30 | .000 | .000 | -0.53 |
|  | Positive | Neutral | t(114)=-8.88 | .000 | .000 | -0.65 |  | t(114)=-5.88 | .000 | .000 | -0.44 |
| *Note.* Early middle, 35-47 years old. Late middle, 48-59 years old. Older, at least 60 years old. *p*.adj, *p*-values adjusted for the false discovery rate (FDR). *d*, effect size measured by Cohen’s *d*. | | | | | | | | | | | |

## Table S11

*Descriptive Statistics of Gist and Specific Memory Trade-Off for Early Middle-Aged, Late Middle-Aged, Older Adults, and the Total Sample*

|  |  | **Gist Trade-Off** | | **Specific Trade-Off** | |
| --- | --- | --- | --- | --- | --- |
|  |  | *M* | *SD* | *M* | *SD* |
| Negative scenes | Early middle | 0.26 | 0.23 | 0.27 | 0.25 |
|  | Late middle | 0.29 | 0.22 | 0.30 | 0.26 |
|  | Older | 0.21 | 0.25 | 0.27 | 0.27 |
|  | **Total** | **0.24** | **0.24** | **0.28** | **0.26** |
| Positive scenes | Early middle | 0.29 | 0.23 | 0.26 | 0.22 |
|  | Late middle | 0.28 | 0.22 | 0.22 | 0.27 |
|  | Older | 0.26 | 0.24 | 0.27 | 0.26 |
|  | **Total** | **0.27** | **0.23** | **0.25** | **0.25** |
| Neutral scenes | Early middle | 0.04 | 0.25 | 0.00 | 0.23 |
|  | Late middle | 0.04 | 0.22 | 0.04 | 0.24 |
|  | Older | -0.01 | 0.21 | 0.04 | 0.22 |
|  | **Total** | **0.02** | **0.23** | **0.03** | **0.22** |
| *Note.* Early middle, 35-47 years old. Late middle, 48-59 years old. Older, at least 60 years old. | | | | | |

## Table S12

*Analysis of Variance Test Results for Gist and Specific Memory Trade-Off Effects*

|  | **Age as a categorical predictor** | | | |  | **Age as a continuous predictor** | | | |
| --- | --- | --- | --- | --- | --- | --- | --- | --- | --- |
|  | *F* | *p* | *p.*adj | *ηp2* |  | *F* | *p* | *p.*adj | *ηp2* |
| ***Gist Memory Trade-Off*** | | | | |  |  | | | |
| Age | F(2, 268)=2.49 | .085 | .280 | 0.02 |  | F(1, 270)=2.34 | .128 | .447 | 0.01 |
| Valence | F(2, 536)=136.41 | .000 | .000 | 0.34 |  | F(2, 540)=5.79 | .003 | .023 | 0.02 |
| Delay | F(1, 268)=1.84 | .176 | .308 | 0.01 |  | F(1, 270)=0.10 | .755 | .755 | 0.00 |
| Age x Valence | F(4, 536)=0.53 | .713 | .897 | 0.00 |  | F(2, 540)=0.44 | .647 | .755 | 0.00 |
| Age x Delay | F(2, 268)=0.11 | .897 | .897 | 0.00 |  | F(1, 270)=0.41 | .525 | .755 | 0.00 |
| Valence x Delay | F(2, 536)=2.13 | .120 | .280 | 0.01 |  | F(2, 540)=0.29 | .752 | .755 | 0.00 |
| Age x Valence x Delay | F(4, 536)=0.30 | .880 | .897 | 0.00 |  | F(2, 540)=0.30 | .738 | .755 | 0.00 |
| ***Specific Memory Trade-Off*** | | | | |  |  | | | |
| Age | F(2, 268)=0.20 | .822 | .904 | 0.00 |  | F(1, 270)=1.38 | .241 | .843 | 0.01 |
| Valence | F(2, 536)=116.24 | .000 | .000 | 0.30 |  | F(2, 540)=7.13 | .001 | .006 | 0.03 |
| Delay | F(1, 268)=3.58 | .060 | .210 | 0.01 |  | F(1, 270)=0.04 | .836 | .885 | 0.00 |
| Age x Valence | F(4, 536)=1.45 | .216 | .504 | 0.01 |  | F(2, 540)=0.12 | .885 | .885 | 0.00 |
| Age x Delay | F(2, 268)=0.42 | .658 | .904 | 0.00 |  | F(1, 270)=0.40 | .529 | .885 | 0.00 |
| Valence x Delay | F(2, 536)=0.98 | .377 | .660 | 0.00 |  | F(2, 540)=0.24 | .790 | .885 | 0.00 |
| Age x Valence x Delay | F(4, 536)=0.26 | .904 | .904 | 0.00 |  | F(2, 540)=0.35 | .704 | .885 | 0.00 |
| *Note.* Mixed-model ANOVA with a 3 (Age: Early Middle Age, Late Middle Age, Older Adulthood) x 3 (Scene Valence: Negative, Neutral, Positive) x 2 (Delay: Sleep, Wake) design, and linear mixed-effects ANOVA treating age as a continuous variable were performed for gist and specific memory trade-off effects separately. Early middle, 35-47 years old. Late middle, 48-59 years old. Older, at least 60 years old. *p*.adj, *p*-values adjusted for the false discovery rate (FDR). *ηp^2^*, effect size measured by partial eta-squared. | | | | | | | | | |

## Table S13

*Pairwise T-Tests of Age and Scene Valence for Gist and Specific Memory Trade-Off Effects*

|  |  |  | **Gist Trade-Off** | | | |  | **Specific Trade-Off** | | | |
| --- | --- | --- | --- | --- | --- | --- | --- | --- | --- | --- | --- |
|  |  |  | *t* | *p* | *p*.adj | *d* |  | *t* | *p* | *p*.adj | *d* |
| *Comparing early middle, late middle, and older age* | | | |  |  |  |  |  |  |  |  |
| Negative scenes | Middle1 | Middle2 | t(154)=-0.90 | .372 | .372 | -0.14 |  | t(150)=-0.85 | .397 | 1.000 | -0.14 |
|  | Middle1 | Older | t(190)=1.47 | .144 | .288 | 0.21 |  | t(191)=0.02 | .981 | 1.000 | 0.00 |
|  | Middle2 | Older | t(165)=2.35 | .020 | .059 | 0.35 |  | t(157)=0.89 | .373 | 1.000 | 0.13 |
| Positive scenes | Middle1 | Middle2 | t(156)=0.07 | .945 | 1.000 | 0.01 |  | t(139)=1.05 | .296 | .630 | 0.17 |
|  | Middle1 | Older | t(187)=0.81 | .420 | 1.000 | 0.11 |  | t(196)=-0.26 | .797 | .797 | -0.04 |
|  | Middle2 | Older | t(166)=0.73 | .466 | 1.000 | 0.11 |  | t(150)=-1.26 | .210 | .630 | -0.19 |
| Neutral scenes | Middle1 | Middle2 | t(157)=0.10 | .919 | .919 | 0.02 |  | t(151)=-1.12 | .265 | .573 | -0.18 |
|  | Middle1 | Older | t(166)=1.49 | .137 | .411 | 0.22 |  | t(179)=-1.31 | .191 | .573 | -0.19 |
|  | Middle2 | Older | t(150)=1.42 | .158 | .411 | 0.21 |  | t(144)=-0.01 | .992 | .992 | 0.00 |
| *Comparing negative, positive, and neutral scenes* | | | |  |  |  |  |  |  |  |  |
| Early middle | Negative | Positive | t(85)=-1.14 | .258 | .258 | -0.13 |  | t(85)=0.49 | .622 | .622 | 0.06 |
|  | Negative | Neutral | t(85)=7.06 | .000 | .000 | 0.88 |  | t(85)=8.65 | .000 | .000 | 1.14 |
|  | Positive | Neutral | t(85)=8.05 | .000 | .000 | 1.00 |  | t(85)=9.24 | .000 | .000 | 1.14 |
| Late middle | Negative | Positive | t(72)=0.17 | .862 | .862 | 0.02 |  | t(72)=2.79 | .007 | .007 | 0.34 |
|  | Negative | Neutral | t(72)=7.87 | .000 | .000 | 1.12 |  | t(72)=6.83 | .000 | .000 | 1.06 |
|  | Positive | Neutral | t(72)=8.39 | .000 | .000 | 1.11 |  | t(72)=5.43 | .000 | .000 | 0.68 |
| Older | Negative | Positive | t(114)=-1.91 | .059 | .059 | -0.21 |  | t(114)=0.15 | .881 | .881 | 0.02 |
|  | Negative | Neutral | t(114)=7.83 | .000 | .000 | 0.92 |  | t(114)=7.59 | .000 | .000 | 0.93 |
|  | Positive | Neutral | t(114)=10.55 | .000 | .000 | 1.15 |  | t(114)=8.07 | .000 | .000 | 0.93 |
| *Note.* Early middle, 35-47 years old. Late middle, 48-59 years old. Older, at least 60 years old. *p*.adj, *p*-values adjusted for the false discovery rate (FDR). *d*, effect size measured by Cohen’s *d*. | | | | | | | | | | | |

## Table S14

*Correlations Between Age and Key Study Variables*

|  | *r* | *p* | *p*.adj |  | *r* | *p* | *p*.adj |
| --- | --- | --- | --- | --- | --- | --- | --- |
|  | **Valence** | | |  | **Arousal** | | |
| Ratings for negative scenes | -0.04 | .569 | .569 |  | 0.09 | .122 | .183 |
| Ratings for positive scenes | 0.15 | .013 | .039 |  | -0.11 | .070 | .183 |
| Ratings for neutral scenes | 0.10 | .088 | .132 |  | -0.05 | .371 | .371 |
|  | **Gist** | | |  | **Specific** | | |
| Memory for negative objects | -0.03 | .654 | .654 |  | 0.03 | .571 | .685 |
| Memory for negative backgrounds | 0.08 | .206 | .281 |  | 0.00 | .960 | .960 |
| Memory for positive objects | 0.07 | .234 | .281 |  | 0.10 | .094 | .187 |
| Memory for positive backgrounds | 0.10 | .091 | .182 |  | 0.08 | .202 | .303 |
| Memory for neutral objects | 0.12 | .042 | .125 |  | 0.18 | .003 | .019 |
| Memory for neutral backgrounds | 0.19 | .001 | .008 |  | 0.11 | .082 | .187 |
|  | **Gist** | | |  | **Specific** | | |
| Memory trade-off for negative scenes | -0.10 | .113 | .288 |  | 0.03 | .571 | .571 |
| Memory trade-off for positive scenes | -0.03 | .590 | .590 |  | 0.04 | .467 | .571 |
| Memory trade-off for neutral scenes | -0.08 | .192 | .288 |  | 0.08 | .202 | .571 |
| *Note. p*.adj, *p*-values adjusted for the false discovery rate (FDR). | | | | | | | |

## Table S15

*Linear Regression Predicting Memory with Valence and Arousal Ratings*

|  | **Valence Ratings** | | | |  | **Arousal Ratings** | | | |
| --- | --- | --- | --- | --- | --- | --- | --- | --- | --- |
|  | ***B*** | ***p*** | ***p*.adj** | ***R^2^*** |  | ***B*** | ***p*** | ***p*.adj** | ***R^2^*** |
| **Gist Memory** |  |  |  |  |  |  |  |  |  |
| Negative objects | -0.05 | .033 | .112 | 0.02 |  | 0.06 | .003 | .016 | 0.03 |
| Negative backgrounds | 0.01 | .580 | .696 | 0.00 |  | 0.01 | .510 | .680 | 0.00 |
| Positive objects | 0.03 | .106 | .283 | 0.01 |  | -0.02 | .218 | .348 | 0.01 |
| Positive backgrounds | 0.00 | .946 | .959 | 0.00 |  | -0.01 | .555 | .696 | 0.00 |
| Neutral objects | -0.01 | .818 | .934 | 0.00 |  | -0.03 | .187 | .348 | 0.01 |
| Neutral backgrounds | 0.03 | .264 | .396 | 0.00 |  | -0.05 | .025 | .101 | 0.02 |
|  |  |  |  |  |  |  |  |  |  |
| **Specific Memory** |  |  |  |  |  |  |  |  |  |
| Negative objects | -0.10 | .000 | .002 | 0.05 |  | 0.09 | .000 | .002 | 0.05 |
| Negative backgrounds | -0.03 | .191 | .348 | 0.01 |  | 0.03 | .046 | .138 | 0.01 |
| Positive objects | 0.03 | .161 | .348 | 0.01 |  | -0.02 | .209 | .348 | 0.01 |
| Positive backgrounds | 0.00 | .959 | .959 | 0.00 |  | -0.02 | .166 | .348 | 0.01 |
| Neutral objects | -0.02 | .377 | .532 | 0.00 |  | -0.05 | .013 | .062 | 0.02 |
| Neutral backgrounds | 0.00 | .933 | .959 | 0.00 |  | -0.08 | .000 | .002 | 0.05 |
| *Note. B,* unstandardized slope coefficients. *p*.adj, *p*-values adjusted for the false discovery rate (FDR). *R^2^*, effect size measured by R-squared. | | | | | | | | | |

## Table S16

*Linear Regression Predicting Memory Trade-Off Effects with Valence and Arousal Ratings*

|  | **Valence Ratings** | | | |  | **Arousal Ratings** | | | |
| --- | --- | --- | --- | --- | --- | --- | --- | --- | --- |
|  | ***B*** | ***p*** | ***p*.adj** | ***R^2^*** |  | ***B*** | ***p*** | ***p*.adj** | ***R^2^*** |
| **Gist Memory Trade-off** |  |  |  |  |  |  |  |  |  |
| Negative scenes | -0.06 | .012 | .059 | 0.02 |  | 0.05 | .030 | .089 | 0.02 |
| Positive scenes | 0.03 | .112 | .255 | 0.01 |  | -0.01 | .563 | .614 | 0.00 |
| Neutral scenes | -0.03 | .149 | .255 | 0.01 |  | 0.02 | .301 | .362 | 0.00 |
|  |  |  |  |  |  |  |  |  |  |
| **Specific Memory Trade-off** |  |  |  |  |  |  |  |  |  |
| Negative scenes | -0.07 | .004 | .046 | 0.03 |  | 0.06 | .015 | .059 | 0.02 |
| Positive scenes | 0.03 | .138 | .255 | 0.01 |  | 0.00 | .860 | .860 | 0.00 |
| Neutral scenes | -0.03 | .293 | .362 | 0.00 |  | 0.03 | .213 | .320 | 0.01 |
| *Note. B,* unstandardized slope coefficients. *p*.adj, *p*-values adjusted for the false discovery rate (FDR). *R^2^*, effect size measured by R-squared. | | | | | | | | | |

## Table S17

*Sleep and Alertness Variables: Descriptive Statistics, Main Effects of Age, and Correlations with Gist Memory*

|  | Middle1 | Middle2 | Older | *F* | *r* |
| --- | --- | --- | --- | --- | --- |
| **Alertness for the study session** | |  |  |  |  |
| Study PVT (ms) | 334.39 (60.75) | 336.43 (49.76) | 341.27 (55.69) | 1.71 | -0.16^**^ |
| Study SSS | 2.33 (1.20) | 2.21 (1.23) | 1.93 (1.07) | 3.14^*^ | 0.04 |
| **Alertness for the test session** | |  |  |  |  |
| Test PVT (ms) | 330.10 (54.45) | 335.44 (49.65) | 344.00 (60.61) | 2.17 | -0.19^**^ |
| Test SSS | 2.08 (1.12) | 2.10 (1.32) | 1.77 (1.07) | 2.46^⁺^ | 0.10 |
| **Sleep averaged across the past three nights before the study session** | | | |  |  |
| Bedtime (hh:mm) | 23:46 | 23:22 | 23:11 | 3.14^*^ | 0.12^⁺^ |
| Wake Time (hh:mm) | 07:10 | 06:45 | 06:57 | 1.38 | 0.04 |
| TST (h) | 7.40 (1.05) | 7.39 (1.31) | 7.78 (1.05) | 3.86^*^ | -0.12^*^ |
| SOL (min) | 18.65 (13.37) | 21.13 (20.73) | 20.64 (18.42) | 0.46 | 0.10^⁺^ |
| # of Awakenings | 1.20 (1.05) | 1.37 (1.33) | 1.68 (1.03) | 4.64^*^ | 0.00 |
| **Sleep quality for the Past Month** | |  |  |  |  |
| PSQI | 5.39 (3.29) | 5.05 (3.49) | 5.04 (3.24) | 0.26 | 0.12^⁺^ |
| *Note.* Mean is displayed for Bedtime and Wake Time, while mean (standard deviation) are displayed for all other variables. *F*, between-subject ANOVA for the effect of age (Early Middle Age, Late Middle Age, Older Adulthood) on each variable. *r*, each variable’s correlation with overall gist memory across all scene components. PVT, the average reaction time on the Psychomotor Vigilance. SSS, the Stanford Sleepiness Scale. TST, total sleep time. SOL, sleep onset latency. # of Awakenings, the number of awakenings in the middle of the night. PSQI, the composite score of the Pittsburgh Sleep Quality Index. Level of significance based on unadjusted, original *p*-values. ^⁺^, <.01. ^*^, <.05. ^**^, <.01. ^***^, <.001. | | | | | |

## Table S18

*Sleep Variables from the Night Between the Study and Test Sessions in the Nighttime Sleep Condition (n=137): Descriptive Statistics, Main Effects of Age, and Correlations with Gist Memory*

|  | Middle1 | Middle2 | Older | *F* | *r* |
| --- | --- | --- | --- | --- | --- |
| Bedtime (hh:mm) | 23:48 | 23:08 | 23:13 | 2.39^⁺^ | 0.10 |
| Wake Time (hh:mm) | 07:04 | 06:44 | 06:50 | 0.56 | 0.03 |
| TST (h) | 7.26 ( 1.07) | 7.59 ( 1.27) | 7.63 ( 1.57) | 1.05 | -0.09 |
| SOL (min) | 19.89 (27.39) | 20.57 (17.56) | 19.04 (15.19) | 0.06 | 0.01 |
| Sleep Quality | 2.36 ( 0.88) | 2.14 ( 0.73) | 2.04 ( 0.84) | 1.89 | 0.01 |
| **In the morning, before the test session** | | |  |  |  |
| Problem getting up | 1.56 ( 0.76) | 1.46 ( 0.61) | 1.47 ( 0.85) | 0.21 | -0.09 |
| Feeling sleepy | 2.09 ( 1.00) | 2.09 ( 0.92) | 1.60 ( 0.90) | 4.55^*^ | 0.08 |
| Wish more sleep | 2.49 ( 1.24) | 2.12 ( 1.25) | 1.86 ( 1.29) | 3.13^*^ | -0.06 |
| Scattered thoughts | 1.61 ( 0.89) | 1.46 ( 0.70) | 1.23 ( 0.60) | 3.54^*^ | -0.07 |
| Moving slowly | 1.47 ( 0.89) | 1.37 ( 0.55) | 1.40 ( 0.73) | 0.17 | 0.02 |
| Minutes to “come to” | 25.29 (62.02) | 17.83 (17.60) | 12.16 (11.41) | 1.55 | -0.08 |
| *Note.* Mean is displayed for Bedtime and Wake Time, while mean (standard deviation) are displayed for all other variables. *F*, between-subject ANOVA for the effect of age (Early Middle Age, Late Middle Age, Older Adulthood) on each variable. *r*, each variable’s correlation with overall gist memory across all scene components. TST, total sleep time. SOL, sleep onset latency. # of Awakenings, the number of awakenings in the middle of the night. Level of significance based on unadjusted, original *p*-values. ^⁺^, <.01. ^*^, <.05. ^**^, <.01. ^***^, <.001. | | | | | |
